# Amplifying Colossal Thermal Expansion of a Martensitic Molecular Crystal through Interlayer Shear‐Induced Side‐Chain Liberation

**DOI:** 10.1002/anie.202415821

**Published:** 2024-11-02

**Authors:** Kyoungtae Hwang, Gwangsik Sin, Minwoo Jang, Yoon Mi Choi, Dohyun Moon, Hyungbum Park, Sang Kyu Park

**Affiliations:** ^1^ Functional Composite Materials Research Center Korea Institute of Science and Technology (KIST) Jeonbuk 55324 Republic of Korea; ^2^ Chemical Analysis Center Korea Research Institute of Chemical Technology (KRICT) Daejeon 34114 Republic of Korea; ^3^ Beamline Department Pohang Accelerator Laboratory (PAL)/POSTECH Pohang 37673 Republic of Korea; ^4^ Department of Mechanical Engineering Incheon National University Incheon 22012 Republic of Korea; ^5^ Department of JBNU-KIST Industry-Academia Convergence Research Jeonbuk National University (JBNU) Jeonbuk 54896 Republic of Korea

**Keywords:** Actuator, Colossal thermal expansion, dynamic crystal, martensitic transition, molecular cooperativity

## Abstract

Molecular crystals capable of colossal thermal expansion (TE) are fascinating owing to their substantial and continuous volume changes and reasonably linear responses to temperature. This makes them promising candidates for micromachine applications. Macroscopic motion is driven by subtle yet cooperative movements of molecules that respond to the thermal motions of dynamic functional units. The study of p‐TIPS‐DSB presented here offers a compelling case highlighting the relationship between the degree of dynamicity of functional units and TE behavior. In its α‐phase, the p‐TIPS‐DSB crystal undergoes an irreversible martensitic transition to the β‐phase, accompanied by significant cooperative interlayer shear. This process substantially enhances the mobility of the side‐chains driven by the increased free volume surrounding them. This nearly doubles the volumetric TE coefficient from 255.3 (10) to 444.9 (32) MK^−1^, particularly in the actuation direction from 175.0 (7) to 291.7 (20) MK^−1^, enabling about 4.5 % elongation/contraction. As demonstrated here, p‐TIPS‐DSB exhibits a decent force density (>1.4×10^7^ N m^−3^) and precise motion control capabilities due to its hysteresis‐free and non‐abrupt TE nature. Furthermore, we demonstrated the limited operating distance of colossal TE materials can be amplified by utilizing levers, highlighting the high potential of these materials for use in micromachines.

## Graphical abstract

A novel colossal thermal expansion (TE) material, p‐TIPS‐DSB, amplifies TE behavior via an irreversible α→β martensitic transition. The transition, accompanied by substantial interlayer shear, increases free volume surrounding TIPS side‐chains, significantly enhancing their motility. This mechanism nearly doubles the volumetric TE coefficient in the β‐phase, from 255.3 to 444.9 MK^−1^.

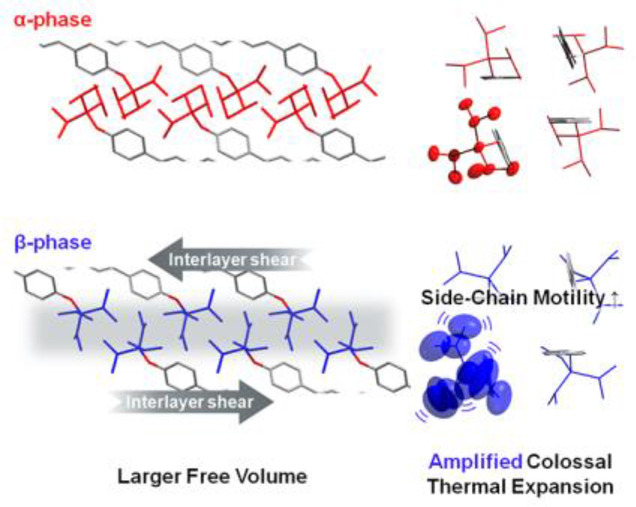

## Introduction

Cooperativity is the fundamental mechanism behind the actuation of dynamic molecular crystals, converting external energy into mechanical motion.^[1]^ During the energy interconversion process, the external energy modifies the conformation or configuration of molecules and their dynamic behavior, playing a pivotal role in triggering collective molecular motion.[^2^, ^3^, ^4^, ^5^] Here, the movements of individual molecules are intrinsically minuscule and typically occur at the (sub−) angstrom scale. However, the ensemble of motions demonstrated by numerous individual molecules within a crystal amplifies the motion of the crystal on a macroscopic scale. This molecular cooperativity underlies beneficial actions in dynamic crystals like contraction/expansion,[^6^, ^7^, ^8^, ^9^] bending,[^10^, ^11^] and twisting.^[12]^ In addition, the (solution−) processability, softness, and chemical versatility of these materials present significant potential for their incorporation into micromachines, such as soft robots, artificial muscles, and microelectromechanical systems in the future.[^13^, ^14^, ^15^]

Thermosalient or thermoelastic crystals are a class of dynamic crystals currently receiving significant attention. This can be ascribed to their rapid and substantial deformations, typically achieving a maximum elongation/contraction of ±3–10 %, and in some exceptional cases, even approaching 50 %.^[9]^ These intriguing attributes arise from their martensitic transitions, which achieve significant lattice shear through the rapid and cooperative molecular rearrangement. Hence, in their role as actuators, thermosalient crystals can deliver a potent stroke to the target, characterized by a high force density and work capacity. However, the first‐order nature of these transitions leads to abrupt volume changes and substantial thermal hysteresis, making them less suitable for miniaturized linear motors that require continuous and precise displacement control.

Hence, this study considers materials with colossal thermal expansion (TE), demonstrating continuous yet substantial volume changes with a reasonably linear response to temperature. Unlike typical crystalline materials with TE coefficients below 20 MK^−1^, some noteworthy materials have TE coefficients exceeding 100 MK^−1^.[^6^, ^9^, ^16^, ^17^, ^18^, ^19^, ^20^, ^21^, ^22^, ^23^, ^24^, ^25^, ^26^, ^27^, ^28^, ^29^, ^30^, ^31^] These materials are referred to as *colossal*, a term originally used for cases where axial TE coefficients exceed 100 MK^−1^,^[26]^ and later expanded to encompass volumetric TE coefficients as well.[^20^, ^24^] Their substantial TE coefficients are attributed to the collective reorientation of molecules, triggered by significant molecular vibration and disordering motion. The strength and pattern of the intermolecular interaction network determine the retention of the packing motif upon expansion and the directionality of expansion, respectively. For example, in an (S,S)‐octa‐3,5‐diyn‐2,7‐diol crystal with a flexible hydrogen bonding network, thermal vibrations promote the cooperative tilting of molecules, resulting in a colossal TE in the tilting direction.^[18]^ Meanwhile, crystals of 1,4‐bis ((E)‐(4‐iodophenyl) diazenyl) benzene forming 2D halogen‐bonded sheets showed nearly zero TE within these sheets.^[16]^ However, they exhibited colossal TE along the sheet normal, stemming from the collective molecular reorganization triggered by the thermally induced pedal motion of the azo units. Incorporating units capable of extensive vibration or dynamic disordering can be considered a design strategy for obtaining materials with significant TE, as demonstrated in above examples.

Herein, we introduce an intriguing dynamic crystalline material, 1,4‐bis ((E)‐4‐((triisopropylsilyl) oxy) styryl) benzene (p‐TIPS‐DSB). This material features dynamic and reconfigurable triisopropylsilyl (TIPS) side‐chains that promote an irreversible thermosalient transition from the metastable α‐phase to the thermodynamic β‐phase, transforming into an amplified colossal TE material with the volumetric thermal expansion coefficient (*α_V_*) reaching 444.9 (32) MK^−1^. After the thermosalient transition during the initial heating, which resulted in a length change of +4.5 %, the crystals could expand to the same length in successive cycles. The *α_V_* of p‐TIPS‐DSB is exceptional compared to that of other organic crystals, which have an estimated mean *α_V_* of 168.8 MK^−1^,^[32]^ and is on par with the highest reported values among organic crystals studied for their thermal expansion. Another important aspect is that this material undergoes the monotropic martensitic transition, allowing structurally similar phases to coexist over a broad temperature range. This makes it an optimal system for studying molecular‐level mechanisms that account for differences in thermal expansion due to variations in local molecular environments. Attributed to this advantage, we could reveal that the amplification of *α_V_* arises from the significant interlayer shear during the α→β transition, granting the dynamic TIPS side‐chains greater freedom. As the temperature increases, the TIPS units undergo continuous enhancement in vibrational motion and conformational reconfiguration, resulting in adaptive lattice expansion. While our study unraveled this mechanism through comprehensive experiments and simulations, we also demonstrated the potential of this crystal to function as a linear motor. We demonstrated the capability of the p‐TIPS‐DSB actuator to modulate its length continuously and relatively linearly, perform work with a high force density, and amplify its operation length using a lever. This study highlights the potential of colossal TE materials as cooperative molecular machines for performing elaborate tasks.

## Results and Discussion

**Figure 1:**
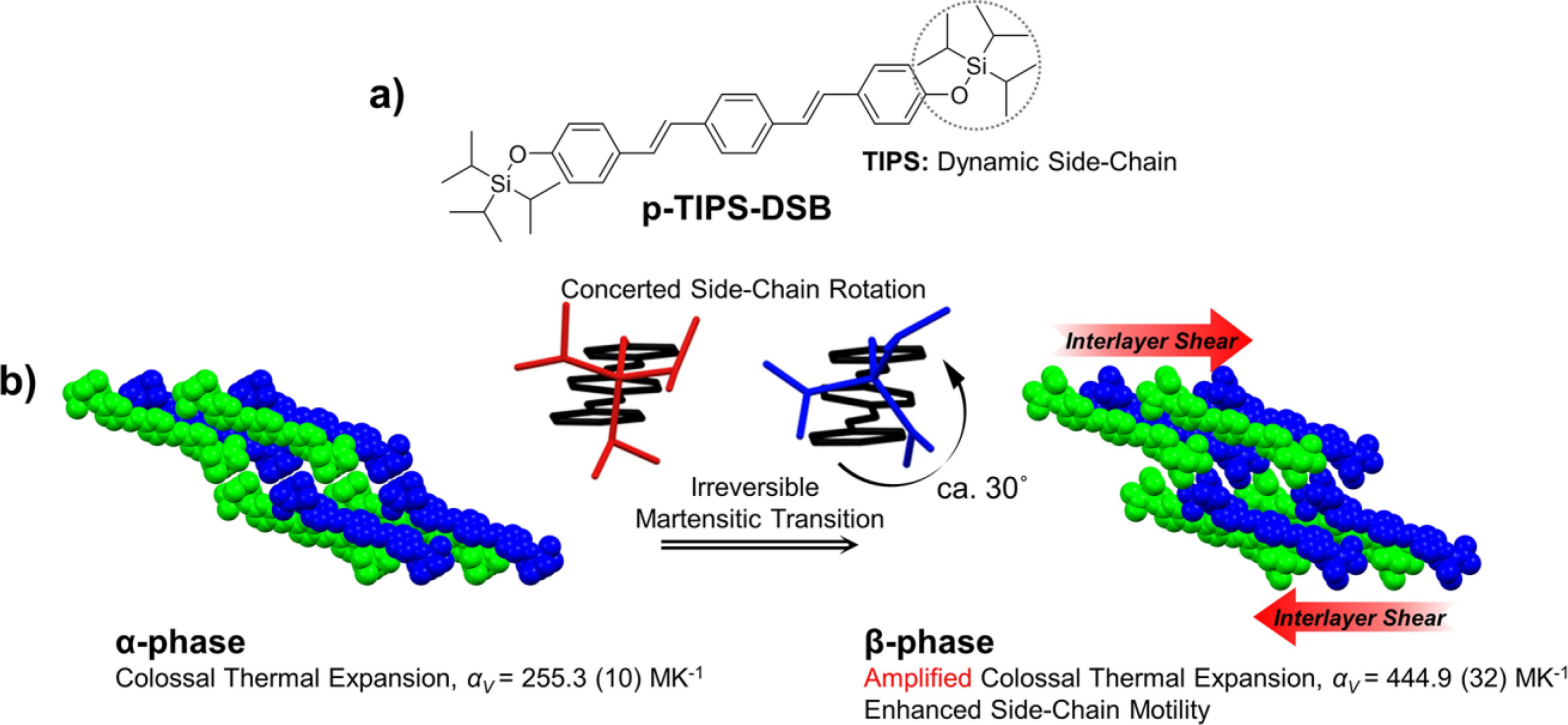
Molecular structure and phase transition mechanism. a) Molecular structure of p‐TIPS‐DSB. b) Schematic description of the mechanism for the martensitic α→β transition, illustrating concerted side chain rotation triggering interlayer shear.

p‐TIPS‐DSB, as outlined in Figure 1a, was synthesized by the procedure detailed in the Supporting Information. This molecule features a distyrylbenzene (DSB) as its backbone, serving as a stator in its crystal structure. Integrating TIPS side‐chains into this stator unit, a dynamic component of particular interest in our recent research, extends its functionality. Due to the presence of both stator and dynamic substituents, one might draw parallels with the molecular rotors studied by Garcia‐Garibay and co‐workers;[^33^, ^34^] however, the p‐TIPS‐DSB crystal exhibited distinct behaviors. The dynamic functions of the TIPS units reported by us can be categorized as follows: 1) initiating martensitic transitions through order‐to‐disorder rotational motions[^35^, ^36^, ^37^] and 2) mitigating the interfacial stress at the twinning boundary during ferroelastic transitions through adaptive reconfiguration.^[38]^ Furthermore, this study revealed new functions of TIPS units: 3) undergoing a single, concerted/adaptive rotation to trigger a thermosalient transition and 4) enabling extensive thermal vibration and collective conformational changes (i.e., reconfiguration) leading to colossal TE. As scrutinized below, the close packing limits the movement of dynamic TIPS units in the initial metastable α‐phase. However, as the temperature rises, their vibrational motion gradually increases until a critical temperature is reached. At this point, a concerted side‐chain rotation of about 30° is achieved, leading to an irreversible martensitic transition to a thermodynamically stable β‐phase. This irreversible α→β transition is accompanied by significant interlayer shear (Figure 1b), subsequently relaxing the interactions between neighboring TIPS side‐chains (i.e., loosened TIPS packing). Consequently, in subsequent cycles, the vibrational and conformational motion of TIPS becomes highly temperature sensitive, allowing instant adjustment of the intermolecular distances on a larger scale without a phase transition, that is, amplified colossal TE.

**Figure 2:**
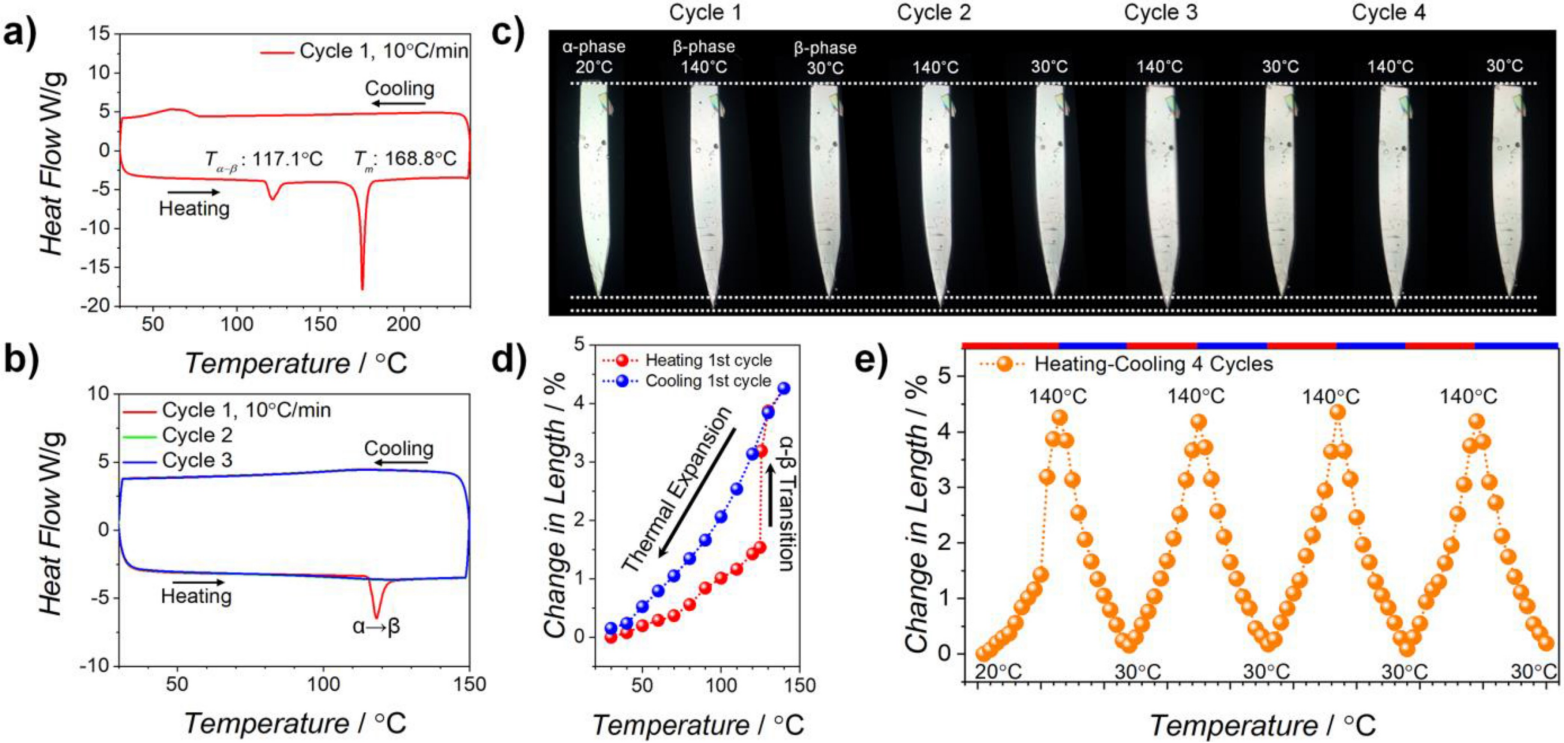
Thermal and thermomechanical behavior. a, b) DSC curves of p‐TIPS‐DSB measured above and below melting temperature. c) VT‐CPOM images of a p‐TIPS‐DSB crystal taken under heating‐cooling cycles. d, e) Change in length of the crystal versus temperature plot measured via VT‐CPOM under the first heating‐cooling cycle, and four consecutive heating‐cooling cycles. The change in length was calculated by (L−L_0_)/L_0_, where L and L_0_ correspond to the length of the crystal and initial length of the α‐phase, respectively.

To understand the thermomechanical and phase transition behaviors, we conducted studies using differential scanning calorimetry (DSC) and variable temperature (VT) cross‐polarized optical microscopy (VT‐CPOM) with high‐quality α‐phase crystals obtained as described in the SI. Two first‐order transitions were observed during heating, with peaks at 117.1 °C and 168.8 °C (Figure 2a), while a broad peak was observed during cooling in the approximate range of 80–50 °C. Using VT‐CPOM (Figure S1a), these were identified as the α→β solid‐state transition, a melting transition, and crystallization, respectively. Closer examination of the VT‐CPOM results proved that the α→β transition shows typical features of martensitic transitions. As shown in Figure S1b and Movie S1, the crystal exhibited a weak but evident thermosalient jump during the transition, characterized by the sudden release of the stored elastic energy, which is a crucial feature of molecular martensites. Furthermore, the transition displayed a phase boundary sweeping behavior, which is another critical characteristic observed in martensitic materials (Figure S1c and Movie S2), indicating that the transition occurred layer‐by‐layer with cooperative molecular motion at the transition interface.

Another intriguing phase transition behavior arises from the cooling stage of the first cycle. As seen in Figure 2b, the temperature range was set below the melting temperature for three cycles. During the initial heating stage, a peak associated with a first‐order transition was detected, resulting in a discontinuous jump in crystal length (Figure 2c and 2d; red spheres). However, no such peaks were observed in the subsequent cycles, including the first cooling stage (Figure 2b). This signifies the irreversibility of the α→β transition, attributed to the metastability of the α‐phase, as elucidated by the density functional theory (DFT) simulations below. Without a first‐order transition, during the first cooling stage, the β‐crystal could contract almost completely to the original length of the α‐crystal (approximately 0.29 % longer than the β‐crystal, as shown in Figure 2d, blue spheres). Throughout subsequent cycles, it consistently expanded and contracted to the same extent solely through TE, displaying continuous and hysteresis‐free characteristics (Figure 2e). It is vital to note that the actuation behavior of the p‐TIPS‐DSB is not limited to four cycles. While the α→β transition may cause minor defects occasionally, the crystals retain their structural integrity in subsequent thermal expansion/contraction cycles.

**Figure 3:**
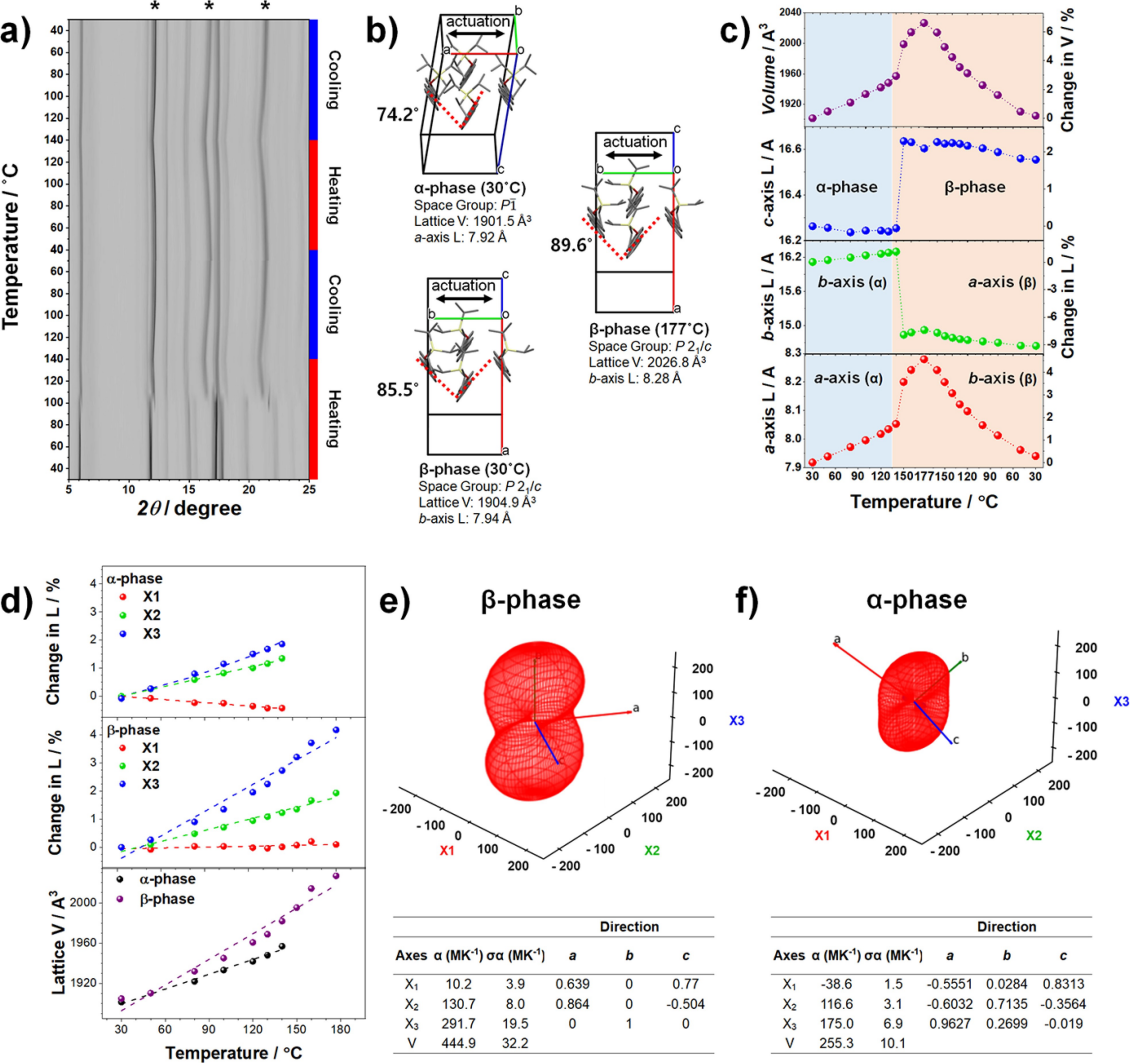
Structure and thermal expansion analyses. a) Contour plot of VT‐PXRD patterns of p‐TIPS‐DSB taken under two heating–cooling cycles. b) Crystal structure, herringbone angle, space group, and important lattice parameters of α‐ (30 °C), β‐ (177 °C), and β‐structures (30 °C). The black arrows indicate the actuation direction, which changes from *a*‐axis to *b*‐axis after the transition. c) Changes in cell length and volume with temperature. Considering the conversion of a‐ and b‐axes after the transition, the a‐ and b‐axes of the β‐phase are represented as b‐ and a‐axes, respectively, in this figure. d) Change in the length of the X_1–3_ and lattice volume. e, f) Expansivity indicatrix and thermal expansion coefficients extracted from PASCal for the β‐ and α‐structures. The unit cells used for these calculations are indicated in Table S2.

To gain further insights into the thermomechanical and phase transition behaviors, we conducted VT powder X‐ray diffraction (VT‐PXRD) and VT‐Raman spectroscopy studies. In the VT‐PXRD (Figure 3a), during the initial heating stage, we observed discontinuous peak shifts around the transition temperature (117.1 °C), confirming the first‐order nature of the α→β transition. This is consistent with the observations shown in Figure 2. We observed continuous yet substantial changes in the positions of the characteristic peaks after α→β (marked by asterisks in Figure 3a), whereas those of the other peaks remained nearly unchanged. This indicates the occurrence of a colossal TE with notable anisotropy. However, using VT‐PXRD alone, we could not assign specific crystallographic planes to these peaks or determine their alignment with actual crystals. Therefore, to accurately extract the crystal length changes and TE coefficients, it is essential to conduct the VT single‐crystal X‐ray diffraction (VT‐SCXRD) study described below.

**Figure 4:**
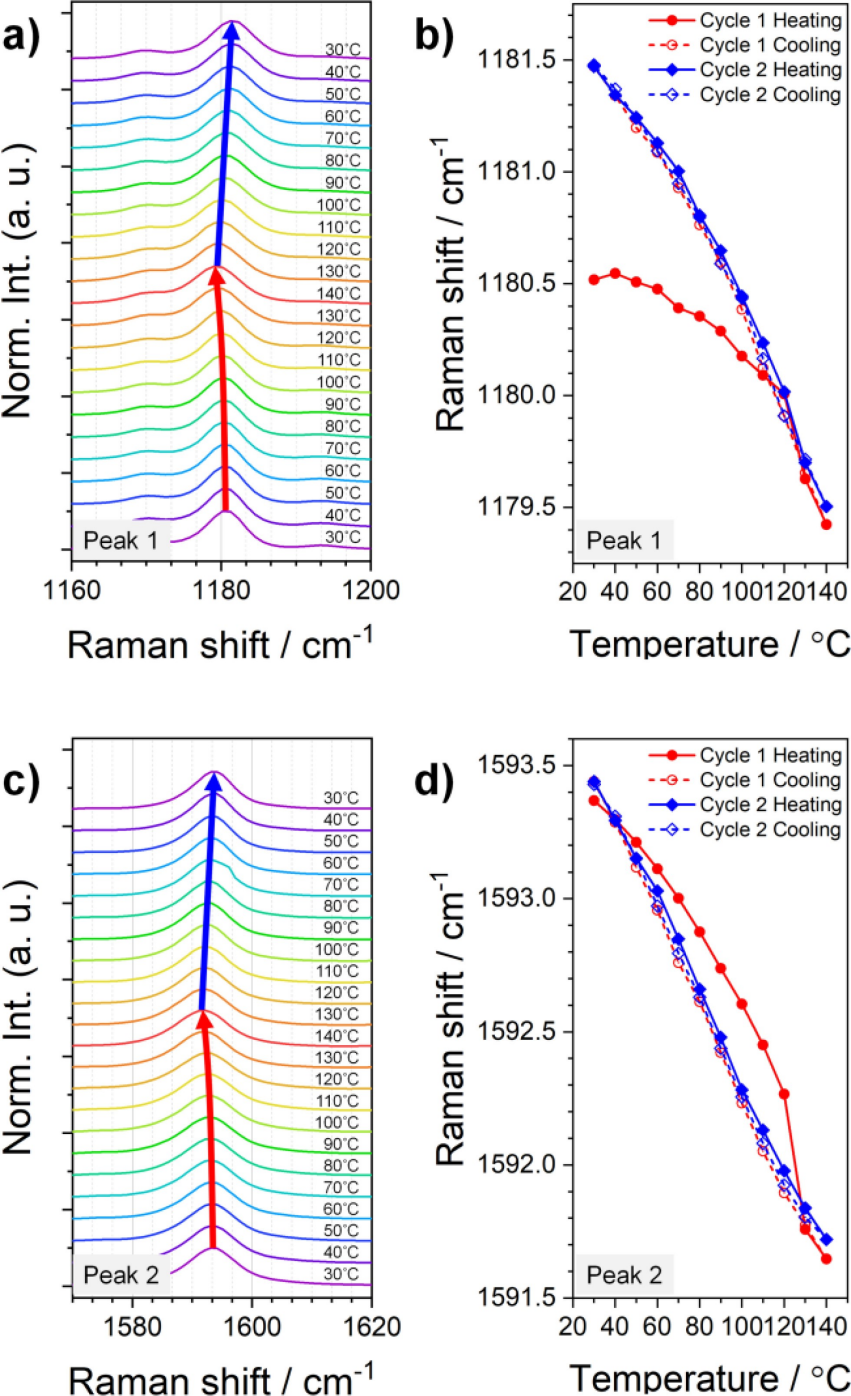
Variable‐temperature Raman analysis. a, b) Change in the Raman frequencies of Peak 1 and c, d) Peak 2 upon temperature alteration. In b) and d), discontinuous jumps in the frequencies and sudden slope changes are evident in the heating stage of the first cycle, which become gradual afterwards.

The VT‐Raman results (Figure 4) excellently agree with the VT‐PXRD findings. The frequency shift of the Raman mode depends primarily on the interatomic bond lengths and molecular conformations. These factors change in coordination with the lattice parameters and intermolecular interactions, making Raman peak shifts a crucial parameter for understanding the phase transition characteristics. Although peak shifts did not occur with equal weighting across all peaks, the changes observed in Raman modes 1 and 2 (Figure S2a) represent the transition characteristics of the p‐TIPS‐DSB crystals. DFT calculations determined that mode 1 corresponds to the vibrations of the side chains and aromatic core, whereas mode 2 corresponds to the stretching of the core unit in the longitudinal direction (Figure S2b, S2c). Figure 4 illustrates the frequency changes of these Raman modes during a heating–cooling cycle (30–140 °C). During the initial heating, a noticeable change in the slope and a sudden jump in the frequency are observed around 120–130 °C (red solid line Figure 4b, 4d), due to the first‐order nature of α→β transition. However, after the transition, a continuous and hysteresis‐free frequency shift occurs during heating and cooling, indicating that the actuation behavior of the β‐crystal is based on TE.

As mentioned above, VT‐SCXRD was conducted to provide comprehensive insights into the phase transitions, thermal expansion, and molecular mechanisms. Table S1 summarizes the structural information observed at both extremes of the heating–cooling process within the range of 30–177 °C (303–450 K), while Table S2 provides a summary of the lattice parameters and assigned phases for all structures obtained from the VT‐SCXRD experiments. Below is an overview of the most crucial findings obtained from the structural analysis on the α‐ and β‐phase, while a more detailed description is available in the Supplementary discussion section of the SI.

1. Due to the displacive and diffusionless nature of the α→β transition, both phases adopt a similar tilted herringbone style; however, the space group changes from $P\bar{1}$ to $P2_{1}/c$ , and the number of symmetrically independent formula unit (Z′) shifts from 1 to 0.5 (Figure 3b).
2. The packing style and symmetry were maintained despite a significant reduction in the lattice volume during cooling from high to room temperature (Figure 3b and 3c). This indicates that the β‐structure is maintained during cooling due to the absence of β→α reverse transition.
3. As indicated in Figure 1b, a significant interlayer shear occurs during the α→β transition. As we unveil below, this becomes the primary cause behind the remarkably enhanced TE of the β‐phase.
4. The habit plane analysis of the α‐phase crystal confirmed that the crystallographic *a*‐axis is parallel to the long axis of the crystal (Figure S3), which correspond to the actuation direction, as shown in Figure 2c–2e. Meanwhile, the *a*‐axis of the α‐phase is transformed into the *b*‐axis of the β‐phase, and thus, the actuation of the β‐phase occurs along its *b*‐axis.

SCXRD analysis reveals that actuation occurs along the *a*‐axis of the α‐phase and the *b*‐axis of the β‐phase. Figure 3c summarizes the changes in the volume and the lengths of the crystallographic axes during heating–cooling, relative to the low‐temperature α‐phase (30 °C). As seen in this Figure, the *a*‐axis of the α‐phase and the *b*‐axis of the β‐phase undergo significant expansion during heating (ΔL=4.57 %), driven by phase transition and thermal expansion, respectively. These changes aligned closely with the observed variation in the crystal length in the VT‐CPOM study (Figure 2c). Interestingly, upon cooling, the *b*‐axis of the β‐phase nearly matches the length of the α‐phase *a*‐axis (ΔL=0.29 %), indicating that the crystal essentially reverts to its original length during the cooling cycle. Meanwhile, the *b*‐ and *c*‐axes of the α‐phase abruptly decreases and increases until it gets transformed, respectively. However, their lengths vary slightly (*b‐*axis: 1.71 %, *c‐*axis: 0.3 %) after the transition to the β‐phase. Therefore, the significant thermal expansion in the volume of the β‐phase is primarily driven by the change in the *b*‐axis, with remarkable anisotropy. This is evident in the close resemblance between the ΔV and ΔL (*a*‐axis) plots in Figure 3c.

Analysis using PASCal revealed that the TE of the p‐TIPS‐DSB crystals is indeed *colossal*. Figure 3d–3 f summarizes the TE coefficients for both α‐ and β‐phases, while Figure. S4 illustrates the alignment of the principal axes (X_1‐3_) within each structure. We first explore the case of β, intriguing for its substantial volume expansion without transition. Figure 3d shows that the volumetric thermal expansion coefficient (*α_V_*) of β‐structure is 444.9 (32) MK^−1^, demonstrating colossal TE. This outstanding *α_V_* value is primarily based on TE along the X_3_ direction (291.7 (20) MK^−1^), which is parallel to the crystallographic *b‐*axis. Additionally, the TE along the X_2_ direction contributed non‐negligibly (130.7 (8) MK^−1^), which approximately aligned with the molecular short‐axis. However, minimal TE is observed in the X_1_ direction (10.2 (4) MK^−1^), aligned with the molecular long‐axis. Meanwhile, although its TE was approximately half that of the β‐phase, the α‐phase also demonstrated colossal TE (*α_V_* =255.3 (10) MK^−1^).

It is worth emphasizing that the TE coefficients should be underestimated because of the overestimation of the temperature. While the α→β transition temperature measured by DSC and VT‐CPOM was approximately 117 °C, VT‐SCXRD recorded it as 150 °C. This difference suggests that the latter method overestimates the temperature. This could be attributed to the quality of the crystal attached to the loop. The phenomenon under investigation may have been influenced by the inherent constraints associated with the *N*_2_ hot‐jet stream‐based temperature controller. Specifically, it is believed that the differences in the distance between the single‐crystal and Cryojet 5 nozzle, along with changes in the ambient temperature, play pivotal roles. Nevertheless, to the best of our knowledge, *α_V_* of 444.9 (32) MK^−1^ in the β‐phase represents one of the highest reported TE values. Moreover, the TE coefficient along the principal axis X_3_ (291.7 (20) MK^−1^) is also comparable to those of the materials showing the highest TE levels (Table S3). According to a study by van der Lee and Dumitrescu,^[32]^ which provides a comprehensive survey of thermal expansion coefficients across a large number of organic materials, p‐TIPS‐DSB ranks 26^th^ out of 4,548 organic crystals with positive *α_V_*, indicating a top‐tier position. However, as the highest TE coefficients reported in their study may include cases where phase transitions were overlooked,^[39]^ crystal structures with different compositions or molecules were used,[^31^, ^40^] or structural refinement errors were present,^[41]^ the actual rank of p‐TIPS‐DSB could potentially have been even higher.

In earlier sections, we delved into the connections between phase transition, thermal expansion, and actuation behavior in p‐TIPS‐DSB under temperature control. Yet, molecular‐level insights into the following critical questions remain unanswered: 1) the irreversibility of the α→β transition, 2) the molecular motions that trigger the α→β transition, and 3) the reason for the enhanced TE observed in the β‐phase. To address these questions, we re‐evaluated the structures comprehensively, coupled with theoretical calculations.

**Figure 5:**
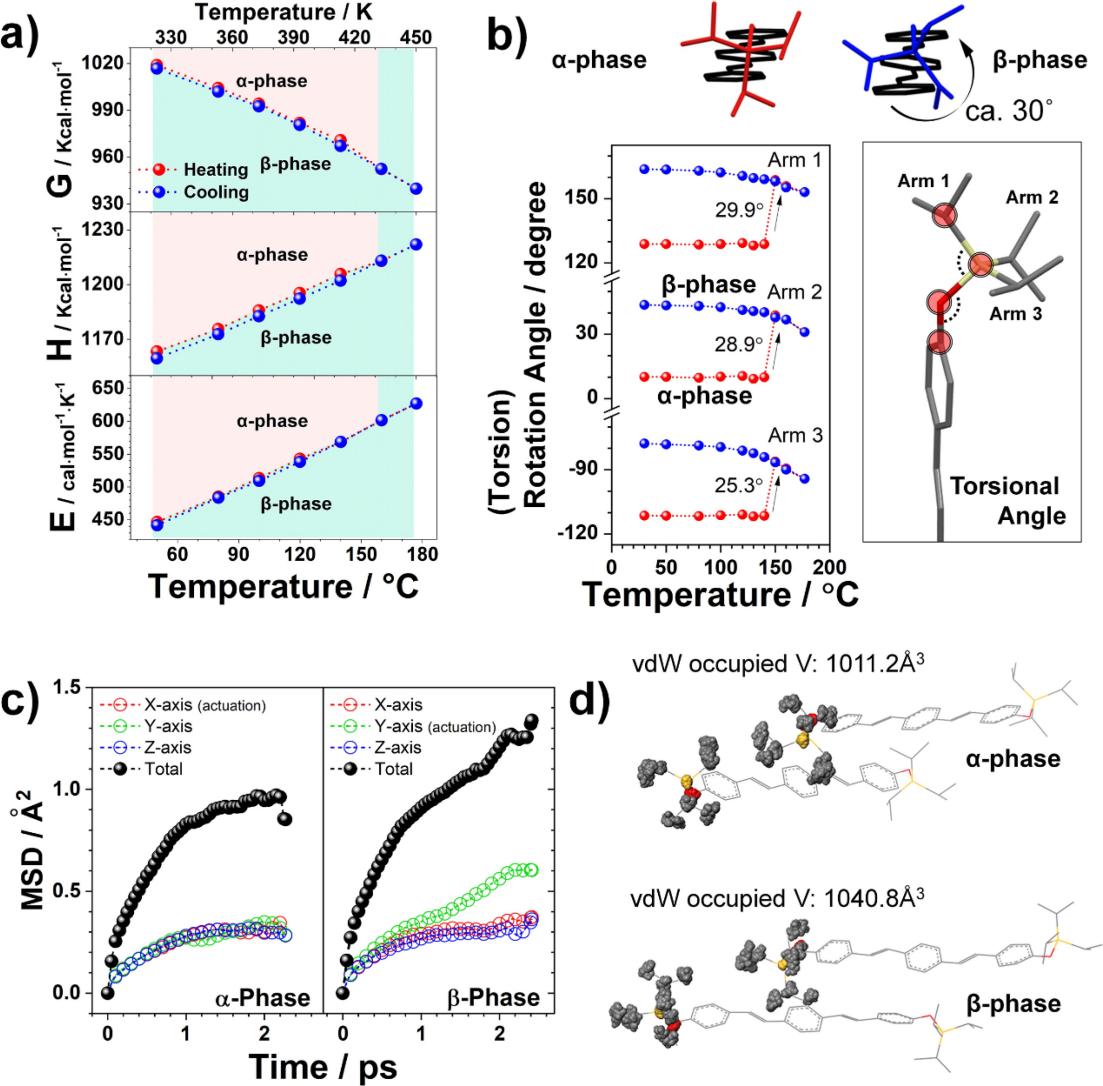
Mechanism for cooperative interlayer shear and amplified thermal expansion behavior. a) DFT‐calculated Gibbs free energy (G), enthalpy (H), and entropy (E) versus temperature plots of the SCXRD structures obtained under heating (red) and cooling (blue). The assigned phase of each structure is indicated by a light red (α) and light blue (β) background. b) Rotation (torsional) angle versus temperature plots measured from three different arms of TIPS unit. c) MD‐simulated mean squared displacement (MSD) for TIPS units of the polymorphs. The β‐phase exhibits particularly large values along the Y‐axis, which corresponds to the actuation direction. d) MD‐simulated van der Waals (vdW) occupied volume for TIPS of the polymorphs at 413 K for 2.25 ps.

We first investigated the reasons behind the irreversibility of the α→β transition using DFT calculations. Figure 5a shows the Gibbs free energy, enthalpy, and entropy plots as function of temperature. In this analysis, we acquired the Gibbs free energies of the resolved structures at different temperatures following sufficient geometric relaxation of the constituent molecules (refer to Supporting Information for simulation details). This plot explicitly shows that the α‐crystal initially obtained is metastable, and the β‐crystal is thermodynamically favored across all temperatures. Therefore, the transition is monotropic within the temperature range of interest. Here, the enthalpy is consistently lower in the β‐phase, with the entropy initially lower before the transition temperature, exhibiting a slight reversal around it. However, even with the lower enthalpy in the β‐phase, we observed that the interlayer cohesive energy, primarily governed by the interaction between neighboring side‐chains, is lower in this phase. This observation supports further discussion on the enhanced TE behavior of the β‐phase, see below.

Following, we deciphered the molecular mechanisms for the α→β phase transition. As described above, the transition essentially maintains the packing style but induces substantial *interlayer shear* (Figure 1b). Here, interlayer shear denotes a pronounced lattice displacement between the vertically stacked layers, causing the molecular layers to shift collectively over a considerable distance comparable to the cell length (see Supporting Information for a detailed description of its rationale (Figure S5, S6). However, considering the steric hindrance induced by the bulky TIPS units that interlock in a zigzag fashion, this shear process was surprisingly remarkable (Figure S7). Therefore, the essence of this phenomenon lies in how it successfully overcomes the significant steric hindrance during the structural transition, and we highlight that the reconfiguration of the side‐chains facilitates the transition. Figure 5b, Figure S8, and Movie S3 show the extent of side‐chain reorientation with respect to temperature. This is particularly evident from Figure 5b. While the side‐chains in the α‐phase maintain their orientation well despite the temperature variations, we could observe an approximate 30° rotation and conformational change in the isopropyl branches immediately following the α→β transition. We surmise that the interlayer shear, which involves relocating the adjacent layer to a new position and transforming it into a structure with higher symmetry, is primarily driven by these side‐chain motions. However, it is essential to note that the corresponding side‐chain motions differ from the cases observed in the thermoelastic transitions of ditert‐butyl[1]benzothieno[3,2‐b][1]1benzothiophene and 6,13‐Bis(triisopropylsilylethynyl)pentacene (TIPS−P), i.e., dynamic rotation.[^35^, ^36^] In this case, instead of toggling between the different conformational states, the side‐chain underwent a single concerted rotation. This behavior resembles the cases observed in the ferroelastic transitions (i.e., deformation twinning) of TIPS−P and its analogs, showing that the adaptive reconfiguration of side‐chains to lower interfacial stress arises in a sterically demanding twinning boundary.^[38]^ Likewise, in the α→β phase transition, it seems that the side‐chains reconfigure to relocate adjacent molecular layers and adaptively alleviate the steric hindrance to overcome the transition barrier.

Meanwhile, the difference in side‐chain motility between the α‐ and β‐phases suggests the reason behind enhanced TE coefficients observed in the β‐phase. Figure 5c shows the ab initio molecular dynamics simulated mean squared displacement (MSD) of side‐chains in the α‐ and β‐phases at 140 °C. The results indicate that the side‐chains in the β‐phase exhibit greater motility, supported by the larger motion of the side‐chains at the same temperature. The simulation results agree with the thermal ellipsoid plots (Figure S9 and Movie S4), presenting a notable enlargement of the ellipsoids, particularly for the TIPS units in the β‐phase at the same temperature. More intriguingly, as shown in Figure 5c, the Y‐axis MSD in the β‐phase increases notably, aligning with the actuation direction of the crystal.

We attribute enhanced TIPS motility in the β‐phase to reduced interaction between the vertically stacked side‐chains, resulting from interlayer shear. Figure S10 presents the calculated cohesive energies for the in‐plane (Pair 1–6) and out‐of‐plane pairs (Pair 7). First, we observed that the cohesive energy of Pair 7 in the α‐phase (−7.24 kcal mol^−1^) is comparable to that of the π–π direction pairs (Pair 1, 4; −7.97 kcal mol^−1^) due to the presence of bulky side‐chains that leads to notable interlayer van der Waals interaction. However, this energy drastically decreases to −3.27 kcal mol^−1^ in the β‐phase and remains relatively stable at −4.15 kcal mol^−1^ even after cooling to 50 °C. This implies enhanced side‐chain motility in the β‐phase due to more relaxed interlayer interactions. Supporting this, the VT‐SCXRD results show significant differences in the −CH⋯ HC− contact distances between the interlayer side‐chains. Figure S11 illustrates the shortest contact distance in the α‐ (30 °C), β‐ (30 °C), and β‐phases (150 °C) as 2.47 Å, 2.59 Å, and 2.75 Å, respectively. These features are reflected in the calculated *d_e_*, that is, the distance from the Hirshfeld surface to the nearest nucleus outside the surface. As presented in Figure S12 and Movie S5, the tightly packed TIPS units in the α‐phase gradually loosen as the temperature rises (changing color from red to yellow in the short *d_e_* region), which become drastically released by the α→β transition (extinction of the red color). However, as the temperature decreased to 303 K, the TIPS units in the β‐phase do not exhibit the same tight packing as those in the α‐phase. This indicates that it has a larger free volume in the β‐phase, as shown in Figure S13. The MD simulation results (Figure 5d) also confirm that the van der Waals occupied volume of side‐chains increases from 1011.24 to 1040.75 Å^3^ in the α‐ and β‐phase, respectively, particularly suggesting a larger free volume around the TIPS in the β‐phase. Another notable aspect of the results is that, despite the increase in free volume, there was no free rotation between the different conformational states. Thus, agreeing with the VT‐SCXRD results, the enhanced thermal expansion is primarily attributed to the enhanced thermal vibration of the side‐chains. This leads to cooperative molecular rotation and displacement, as evidenced by the changes in the herringbone angle (Figure 3b) and *b*‐axis length (Figure 3c), respectively. Based on the cohesive energy, short contact distance, *d_e_*, and free volume analysis, we conclude that the increased side‐chain motility after the interlayer shear upon α→β transition is likely to promote the sensitive thermal responsiveness and volume expansion in the β‐phase, leading to the continuous actuation motion.

**Figure 6:**
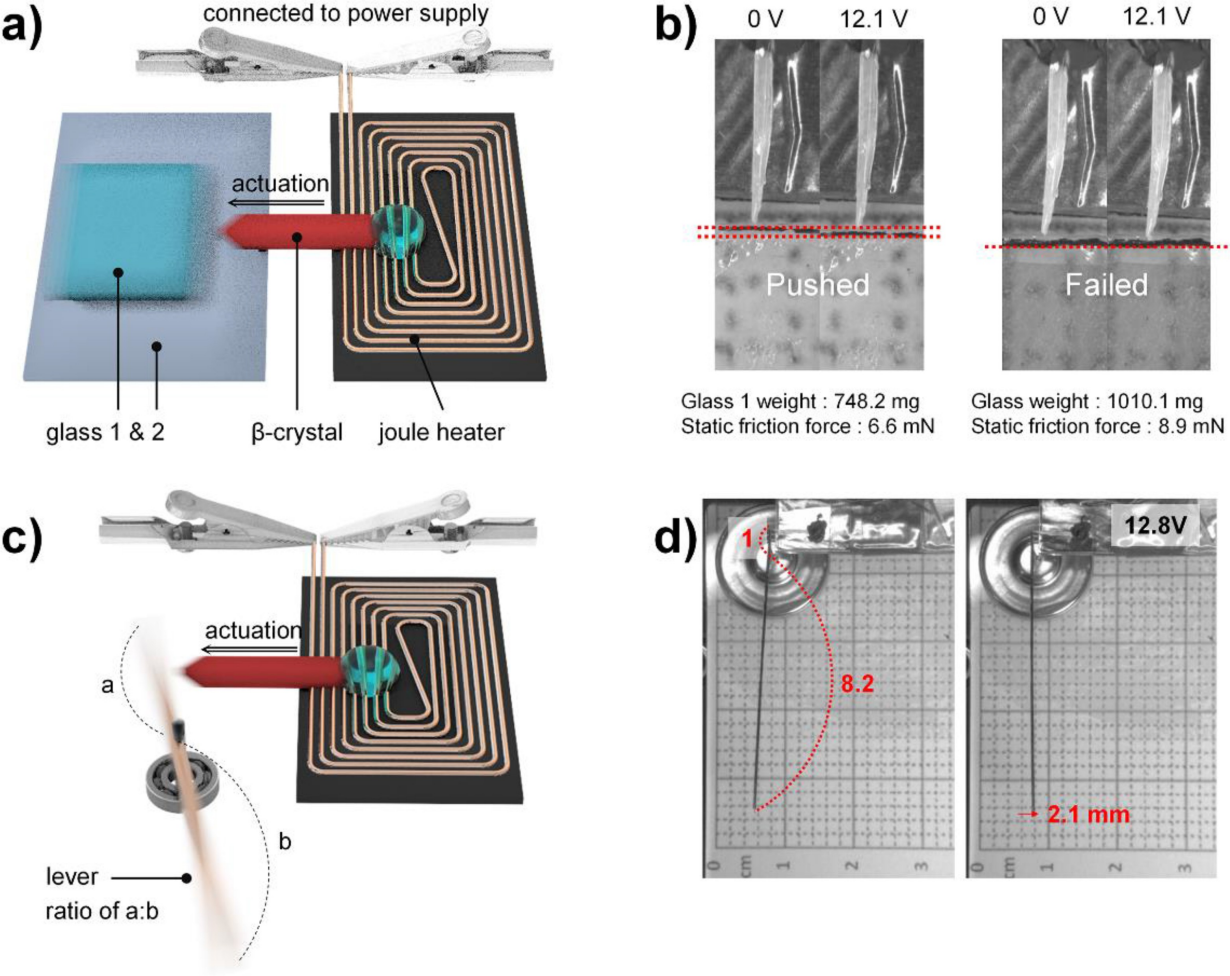
Actuator demonstration. a) Schematic illustration and b) experimental results for evaluating the maximum pushing force exerted by the p‐TIPS‐DSB β‐crystal. c) Schematic illustration and d) experimental results for amplifying the operation distance of the β‐crystal actuator using a lever.

The characteristics of the p‐TIPS‐DSB examined above, including the colossal anisotropic TE and continuous, non‐abrupt elongation capability, indicate the potential utilization of this material in miniaturized linear motors. How much force can this β‐crystal generate or withstand? To validate this, we performed an experiment introduced by Duan et al., which assessed the maximum pushing force exerted by the crystal (equivalent to the maximum static friction between two clean glass plates that the actuating crystal could overcome without breaking), as shown in Figure 6a).^[42]^ As illustrated in Figure 6b, and Movies S6 and S7, the crystal (weight: 0.52 mg, volume: 475.4 mm^3^) could generate pushing forces of ca. 6.6–8.9 mN, which corresponds to ca. 1.4–1.8×10^7^ N m^−3^ in force density. The force density values fall within the range reported for thermosalient organic crystal actuators, surpassing those of ceramic piezoactuators and electrostatic actuators.^[9]^ Meanwhile, concerns may arise regarding the practicality of employing such colossal TE materials as actuators for shorter operating distances than thermosalient materials. However, we demonstrate that the operating distance can be easily amplified using mechanical components like levers. We utilized a lever made from a PET sheet, as shown in Figure 6c, with a leverage ratio of 1 : 8.2, to amplify the operating distance by a factor of 8.2 (also see Movie S8 for actuator motion, amplified by a 1 : 15 lever). Upon applying 12.8 V to the Joule heater beneath the crystal, the crystal elongated from 5.75 mm to 6.04 mm, resulting in a 0.29 mm (5.05 %) increase in length (Figure 6d). Simultaneously, the lever moved a distance of approximately 7.24 times longer (2.1 mm), which corresponds to 36.5 % of the crystal‘s length. Although the extent of elongation is crucial for evaluating dynamic crystal actuators, we emphasize that an excellent operating distance is not indispensable because it can be easily amplified through device design. Instead, factors such as continuity, linearity, and force density are more critical for linear motor applications, as demonstrated by the p‐TIPS‐DSB. This study highlighted the potential of colossal TE materials, showcasing their ability to function as cooperative molecular machines to perform elaborate tasks.

## Conclusions

The p‐TIPS‐DSB crystal developed in this study can be considered an ideal candidate for elucidating the interplay between the thermal expansion behavior and dynamicity of functional units. The structures of α‐ and β‐phases closely resemble each other but coexist in the temperature range of our interest, due to the irreversibility of the α→β transition, which is martensitic. Additionally, the free volume surrounding the dynamic TIPS side‐chains differed significantly; therefore, this system underscores the impact of the motility of dynamic functional units on thermal expansion. As we unravel through comprehensive structural analysis combined with simulations, the free volume surrounding TIPS side‐chains increases in the β‐phase, primarily due to the cooperative interlayer shear. This led to enhanced side‐chain motility, particularly in the actuation direction, for which we pinpointed the molecular‐level origin of the amplified TE behavior observed in the β‐phase.

The anisotropic and enhanced side‐chain motion in the β‐phase facilitates cooperative molecular reorganization on a larger scale. Indeed, the α_*V*_ increased from 255.3 (10) to 444.9 (32) MK^−1^ through the α→β transition. Furthermore, once p‐TIPS‐DSB achieves the thermodynamically stable β‐phase, there is no longer an abrupt first‐order transition, allowing for continuous and hysteresis‐free elongation/contraction of approximately 4.5 %. Moreover, the fact that the motion operates at a force density surpassing 1.4×10^7^ N m^−3^ suggests that colossal TE materials, such as p‐TIPS‐DSB, hold significant promise as cooperative molecular machines capable of precise motion control.

## Supporting Information

Supporting Information is available from the Wiley Online Library or from the author.

## Conflict of Interests

The authors declare no conflict of interest.

1

## Supporting information

As a service to our authors and readers, this journal provides supporting information supplied by the authors. Such materials are peer reviewed and may be re‐organized for online delivery, but are not copy‐edited or typeset. Technical support issues arising from supporting information (other than missing files) should be addressed to the authors.

Supporting Information

Supporting Information

Supporting Information

Supporting Information

Supporting Information

Supporting Information

Supporting Information

Supporting Information

Supporting Information

## Data Availability

The data that support the findings of this study are available in the supplementary material of this article.
