# Supplementary material for: Amplifying Colossal Thermal Expansion of a Martensitic Molecular Crystal through Interlayer Shear‐Induced Side‐Chain Liberation
Source: Angew Chem Int Ed Engl. 2024 Nov 2;64(4):e202415821. doi: 10.1002/anie.202415821 (PMC11753603; doi:10.1002/anie.202415821)
Supplement: Supplementary file 1 — Supporting Information [file ANIE-64-e202415821-s006.pdf]

Angewandte Chemie
Eine Zeitschrift der Gesellschaft Deutscher Chemiker

Supporting Information

Amplifying Colossal Thermal Expansion of a Martensitic Molecular Crystal through Interlayer Shear-Induced Side-Chain Liberation

K. Hwang, G. Sin, M. Jang, Y. Mi Choi, D. Moon, H. Park*, S. Kyu Park**

Supporting Information

Amplifying Colossal Thermal Expansion of a Martensitic Molecular Crystal through Interlayer Shear-induced Side-chain Liberation

Kyoungtae Hwang,[a] Gwangsik Sin,[a] Minwoo Jang,[a] Yoon Mi Choi,[b] Dohyun Moon,[c*] Hyungbum Park,[d*] Sang Kyu Park,[a,e*]

[a] K. Hwang, G. Sin, M. Jang, S.K. Park
Functional Composite Materials Research Center
Korea Institute of Science and Technology (KIST)
Jeonbuk 55324, Republic of Korea
E-mail: skpark86@kist.re.kr

[b] Y. M. Choi
Chemical Analysis Center
Korea Research Institute of Chemical Technology (KRICT)
Daejeon 34114, Republic of Korea

[c] D. Moon
Beamline Department
Pohang Accelerator Laboratory (PAL)/POSTECH
Pohang 37673, Republic of Korea
E-mail: dmoon@postech.ac.kr

[d] H. Park
Department of Mechanical Engineering
Incheon National University
Incheon 22012, Republic of Korea
E-mail: gamamle@inu.ac.kr

[e] S. K. Park
Department of JBNU-KIST Industry-Academia Convergence Research
Jeonbuk National University (JBNU)
Jeonbuk 54896, Republic of Korea

Table of Contents

Methods ········· **Pg. 1**

Synthesis ········· **Pg. 3**

Simulation ········· **Pg. 4**

Supplementary Discussion ········· **Pg. 5**

Supplementary Table ········· **Pg. 8**

Supplementary Figures ········· **Pg. 15**

Captions for Supplementary Movies ········· **Pg. 26**

References ········· **Pg. 27**

Methods

Materials

Tetrahydrofuran (anhydrous, ≥99.9%, Sigma-Aldrich), 4-hydroxybenzaldehyde (98%, Sigma-Aldrich), N,N-dimethylformamide (anhydrous, 99.8 %, Sigma-Aldrich), imidazole (99%, Alfa Aesar), potassium tert-butoxide (t-BuOK, 97%, Alfa Aesar), triisopropylsilyl chloride (TIPS-Cl, > 95%, TCI), and tetraethyl p-Xylylenediphosphonate (> 95%, TCI) were purchased and used as received for the synthesis of p-TIPS-DSB. For actuator fabrication, PELCO® colloidal graphite (isopropanol base, Ted Pella Inc.) was used to attach the p-TIPS-DSB single crystals to heating elements.

General methods

To characterize the synthesized product, ^{1}H- and ^{13}C-NMR spectra were obtained using an Agilent NMR 600 MHz spectrometer. The mass spectrum of p-TIPS-DSB was obtained using a MALDI-TOF/TOF™ 5800 system. Differential scanning calorimetry was conducted using a TA Instruments Q20. To observe the transition and thermal expansion behaviors of p-TIPS-DSB crystals, we employed a polarized optical microscope (Eclipse LV100N POL, Nikon) equipped with a high-speed camera (DS-Fi3, Nikon) and a temperature control stage (LTS420, Linkam Scientific). Variable-temperature (VT) Raman spectra were obtained using an InVia Raman microscope with 1200 g/mm grating (785nm laser, Renishaw Corporate, UK), where the temperature was controlled using the LTS420 stage. VT powder X-ray diffraction (VT-PXRD) patterns were collected using a PANalytical X'Pert PRO diffractometer with Cu Kα radiation. The d_e mapping on the Hirshfeld surface of the p-TIPS-DSB structures was simulated using Crystal Explorer 21.5, with the scales uniformly set to 1.0–2.4 Å for all structures.[1] The thermal expansion coefficients of single crystals during the heating–cooling process were calculated using PASCal (www.pascalapp.co.uk).[2]

Crystal growth

The single crystals of p-TIPS-DSB were obtained through the vapor diffusion method. Specifically, vials containing the p-TIPS-DSB solution (5 mg mL^{-1}, THF) were placed in a methanol bath. The bath was tightly sealed using plastic wrap and stored in the dark at room temperature. After growing to a sufficient size, the crystals were filtered and dried using a Buchner funnel and a filter flask.

Habit plane analysis

For the habit plane analysis, the single crystal of p-TIPS-DSB was mounted on a Mitegen loop. SCXRD data for crystal facet indexing was performed on a Rigaku XtaLABSynergy diffractometer with Cu-Kα radiation (λ = 1.54184 Å). The images of the crystal were captured by rotating the φ axis at a speed of 6°/frame. A total of 60 frames of the crystal were recorded by the equipped camera. By combining the crystal lattice information and rotation images of the crystal, the habit planes of the p-TIPS-DSB crystal were determined using CrysAlisPro software.

VT-SCXRD

For the VT-SCXRD experiment of the p-TIPS-DSB crystal, the crystal was directly retrieved from the as-synthesized batch with a Mitegen loop attached to the goniohead, and transferred to a nitrogen stream (303–450 K). The data collection was conducted using a synchrotron-based X-ray source produced from a PLS-II 2D bending magnet with a Si (111) double crystal monochromator (0.70000 Å) and Rayonix MX225HS CCD area detector. The PAL BL2D-SMDC program[3] was used for the collection of one set of data under the following conditions: a detector distance of 66 mm, 1-axis omega scan with $\Delta\omega$ of 3°, and an exposure time of 1 sec/frame. HKL3000sm (ver. 717.6)[4] was used for cell refinement, reduction, and absorption correction. The structures were solved using the intrinsic phasing method using SHELXT-2018/2[5] and refined using full-matrix least-squares on F^2 using SHELXL-2019/3. [6] All non-hydrogen atoms were refined anisotropically, and all H atoms were placed in geometrically idealized positions and constrained to ride on their parent atoms with C—H = 0.93–0.98 Å and with U_{iso} (H) values of 1.2 and 1.5 U_{eq} of the parent atoms. The variable temperature was controlled using an Oxford Instrument Cryojet 5 system, while the occurrence of transitions was monitored *via* a camera during the heating–cooling process of the mounted single crystals. The temperature at which the α-β transition occurred was found to be approximately 145 °C, causing a sudden change in the crystal length. Considering that the transition temperature is approximately 117°C as measured by DSC or VT-CPOM, it has been observed that the temperature readings obtained from VT-SCXRD are somewhat overestimated compared to other methods. The phenomenon in question is thought to be affected by the distance between the single crystal and nozzle of Cryojet 5, as well as the ambient temperature in the experimental hutch. The TwinRotMat routine within the PLATON program suggested a twin law for the 303K_H, 323K_H, 353K_H, and 413K_H crystal data.[7] These structures were re-refined using SHELXL in the HKLF 5 format. The twin law matrix provided, with rows corresponding to each temperature, is as follows: 303K_H: [1 0 0 / 0.069 -1 0 / 0.537 0 -1], with 766 out of 11,333 total reflections overlapping, and a BASF value of 0.48. 323K_H: [1 0 0 / 0.071 -1 0 / 0.541 0 -1], with 767 out of 10,785 total reflections overlapping, and a BASF value of 0.45. 353K_H: [1 0 0 / 0.076 -1 0 / 0.546 0 -1], with 721 out of 9,173 total reflections overlapping, and a BASF value of 0.38. 413K_H: [1 0 0 / 0.107 -1 0 / 0.582 0 -1], with 674 out of 8,282 total reflections overlapping, and a BASF value of 0.18.

Actuator Demonstration

For the demonstration, the single crystals were attached to a Kapton film heater, and the temperature was controlled by adjusting the input power. The PELCO® colloidal graphite (Ted Pella Inc.) was used to fix the single crystal onto the film heater. RDP-303 DC was used to supply power to the Kapton film heater, and the temperature was measured in real-time using a digital thermometer (TC-400A, LINE SEIKI). Using the actuator, we pushed slide glasses with different weights placed on another slide glass to characterize force density. For the experiment that amplifies the operating distance of actuators, we used a lever constructed with a 5 × 40 mm^2 PET sheet mounted on top of a rotator. The movement of the slide glass and lever by the actuator was recorded using a CCD camera (acA1300-60gm, Basler), equipped with MLH-10X lense (computer).

Synthesis

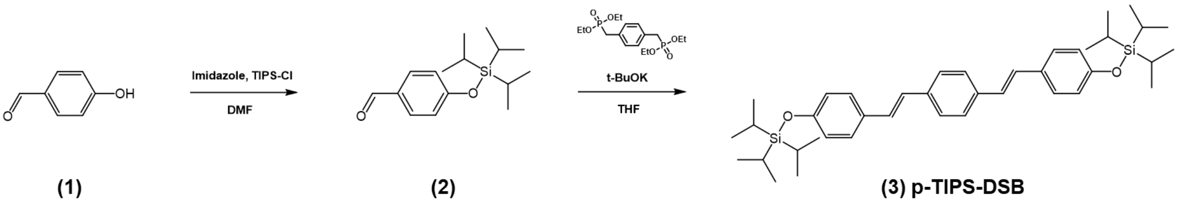

Scheme S1. Synthesis route for obtaining (3) p-TIPS-DSB.

4-((triisopropylsilyl)oxy)benzaldehyde (2):

In a 250 mL two-neck round-bottom flask, 1 g (8.1 mmol) of 4-hydroxybenzaldehyde and 1.33 g (19.6 mmol) of imidazole were dissolved in 25 mL of anhydrous DMF. The mixed solution was cooled to 0 °C in an ice bath. Then, 1.89 g (9.8 mmol) of triisopropylsilyl chloride was slowly added to the solution. The mixture was heated to 60 °C and stirred for 2 h. After cooling to room temperature, the reaction mixture was washed with water, and the organic layer was extracted with ethyl acetate, dried with anhydrous magnesium sulfate ($MgSO_4$), and filtered. The mixture was purified *via* column chromatography (Hexane:ethylene acetate = 9:1). The product, a colorless oil, was obtained after the evaporation of the eluent (1.26 g, 4.5 mmol, yield: 55 %). ^{1}H-NMR (600 MHz, $CDCl_3$, ppm): 9.88 (s, 1H), 7.77 (d, 2H), 6.97 (d, 2H), 1.25-1.32 (m, 3H), 1.10 (m, 18H) ppm.

1,4-bis((E)-4-((triisopropylsilyl)oxy)styryl)benzene ((3), p-TIPS-DSB):

To a 250 mL two-neck round-bottom flask, 2.3 g (8 mmol) of compound (2), 1.25 g (3.3 mmol) of tetraethyl (1,4-phenylenebis(methylene))bis(phosphonate), and 1.11 g (9.9 mmol) of potassium tert-butoxide were dissolved in tetrahydrofuran. Then, the mixture was heated and refluxed at 90 °C for 24 h. After cooling to room temperature, the reaction mixture was washed with water, and the organic layer was extracted with dichloromethane. The mixture was purified *via* flash column chromatography employing dichloromethane as the eluent. The product was recrystallized using a dichloromethane/hexane mixture and then filtered to obtain colorless crystals (1 g, 1.5 mmol, yield: 48 %). ^{1}H-NMR (600 MHz, $CDCl_3$, ppm): 7.45 (s, 4H), 7.38 (d, 4H), 7.04 (d, 2H), 6.94 (d, 2H), 6.86 (d, 4H), 1.26 (m, 6H), 1.11 ppm (m, 36H). ^{13}C-NMR (600 MHz, $CDCl_3$, ppm): 155.92, 136.67, 130.46, 128.13, 127.77, 126.27, 120.15, 17.94, 12.70. MS (MALDI-TOF) (EI, m/z): [M − H]+ calcd for $C_{40}H_{58}O_2Si_2$, 626.40, found, 626.3562.

Simulations

Model preparation and free energy calculation

The density functional theory (DFT) calculations were performed using DMol3 DFT module in Materials Studio 2023 commercial software package. All the DFT calculations were conducted based on the results of experimental structural analyses for reliable theoretical predictions. The unit cells of p-TIPS-DSB were prepared *via* a structural relaxation procedure starting from the initial crystal structures obtained from VT single-crystal X-ray diffraction (VT-SCXRD). Note that the unit cell structures were secured under different temperature and phase conditions (323K-α/β, 353K-α/β, 373K-α/β, 393K-α/β, 413K-α/β, 433K-β, 450K-β) to consider volume variations due to the temperature effect. The structural relaxation procedure consists of iterative geometric optimization and short dynamics (*NVT* canonical ensemble for 50 fs) to fully equilibrate the given atomistic configuration. To identify the convergence of geometric optimization, the energy, maximum force, and maximum displacement criteria were simultaneously adopted with tolerances set to $1.0e^{-5}$ Ha, $2.0e^{-3}$ Ha/Å, and $5.0e^{-3}$ Å, respectively. For all simulations, we adopted the GGA-PBE[8] functional and DNP basis set with long-range dispersion correction suggested by Grimme.[9] The self-consistent field was determined with a convergence criterion of $1.0e^{-5}$ Ha.

The Gibbs free energies for the considered temperatures and phases (323K-α/β, 353K-α/β, 373K-α/β, 393K-α/β, 413K-α/β, 433K-β, 450K-β) were derived from thermodynamic calculations using vibrational analysis. The vibrational analysis provides various translational, rotational, and vibrational energy components by calculating individual vibrational frequencies depending on the temperature.[10] This process ultimately enables the calculation of enthalpy, entropy (with the calculation of the moment of inertia), and Gibbs free energy. The detailed formulation can be found in the literature.[10]

The cohesive energies for several significant non-bonded interactions (Pair 1-7) were calculated based on the equilibrated geometries of 323K- α/β and 433K-β as obtained from the aforementioned procedure. Cohesive energy was determined by subtracting each molecular energy from the total molecular pair energy as below:

$$E_{cohe} = E_{m1+m2} - E_{m1} - E_{m2}, \tag{1}$$

where $E_{m1+m2}, E_{m1},$ and E_{m2} represent the internal energies of the total system, molecule 1 and molecule 2, respectively. Because the consideration of periodicity involves unnecessary bulky interactions while determining cohesive energy, the periodic boundary condition was not considered, thus eliminating the long-range non-bonded interactions.

Ab-initio molecular dynamics (AIMD)

The dynamic behaviors of α- and β- phases were investigated by performing AIMD. All of the simulational conditions are the same as those of the aforementioned static DFT calculations.

The last optimized snapshots of unit cells were determined as initial configurations for dynamics calculations. To elucidate the molecular behaviors in both phases, the dynamic calculations were conducted based on NVT canonical ensemble at 413 K for 2.25 ps with a time step of 1 fs. For consistent temperature control, a massive generalized Gaussian moments thermostat was adopted in this study.

To ascertain the explored volume using the TIPS unit, we employed the general calculation scheme for van der Waals occupied volume.[11] We collected a total of 1,000 positional data snapshots of the TIPS unit during the NVT canonical ensemble simulation for 2.25 ps and consolidated them into a single snapshot, thereby increasing the number of atoms in the cell by 1,000 times. As the scheme calculates the net occupied volume without any overlapping of van der Waals occupied volume, the explored volume of atoms during the dynamics can be efficiently determined.

After obtaining the trajectories from the dynamic calculations, a mean squared displacement (MSD) analysis was performed to elucidate the discrepancy in molecular motility of the TIPS unit between the α- and β-phases. The MSD was calculated by deriving the squared displacement compared to the initial position and averaging it over the considered group of atoms as follows:

$$MSD(t) \equiv \frac{1}{N}\sum_{i=1}^{N}\left|\mathbf{r}(t)-\mathbf{r}(0)\right|^2, \quad (2)$$

where N is the number of atoms, and **r**(t) is the position vector of a certain atom at time t. To separately investigate mobilities along the x, y, and z directions, the mobility of atoms along each x, y, and z direction was investigated by analyzing the MSD for each component of the position vector.

Supplementary Discussion

1. Detailed structural analysis of p-TIPS-DSB polymorphs

As mentioned in the **Results and Discussion** section, we conducted VT-SCXRD studies to gain a more comprehensive understanding of phase transitions, thermal expansion behaviors, and their molecular mechanisms. As this serves as a starting point for the fascinating transition phenomena of p-TIPS-DSB, we first examined the structure of the α-phase. In the α-phase of p-TIPS-DSB, the molecules primarily pack into a herringbone style with a space group of $P\bar{1}$. However, unlike the typical upright herringbone packing, the molecules are significantly tilted with the counter slip angle of 21.28°. We attributed such molecular tilting to the presence of bulky side-chains, as molecules of this type would experience excessive steric hindrance between neighboring side-chains if they were oriented upright. Then, we conducted a habit plane analysis to confirm crystallographic axis alignment in the actual crystal. The analysis of the α-phase crystal (**Figure. S3**) confirmed that the side facets are (0 5 2) and (0 −5 −2). As

these habit planes are parallel to the crystallographic a-axis, it indicates that the long axis of the crystal, i.e., the actuation direction, is also parallel to this axis.

Next, we examined the structure of the β-phase, which emerges as a consequence of the martensitic α→β transition. The martensitic transition is marked by several distinct features, including (1) a close correlation between the pre- and post-transition phases, (2) cooperative molecular motions, (3) a displacive and diffusionless mechanism, (4) retention of neighboring molecules after the transition, and (5) maintenance of structural integrity (in many cases). These characteristics result in a post-transition phase that closely resembles the pre-transition phase, as observed in the β-phase case. The β-phase maintains the herringbone packing structure similar to that of the α-phase, but with a change in the space group to $P2_1/c$; additionally, the *a*- and *b*-axes of α-phase are converted to the *b*- and *a*-axes in β-phase, respectively. Moreover, it has been verified that the crystal retains its herringbone structure with $P2_1/c$ symmetry after cooling back to 30 °C, demonstrating that the β-structure is preserved. See **Table S1** and **2** for the detailed structural parameters of all phases.

2. Details and rationality of the interlayer shear

As we described in the **Results** section, interlayer shear refers to a severe lattice displacement occurring between adjacent layers in the vertical direction, causing the molecular layers to shift a significant distance in a concerted fashion (**Figure. 1b**). To clarify this concept, **Figure. S5** and **6** have been provided. First, it should be noted that herringbone packing has two distinct molecular orientations (**Figure. S5a**). The images presented in **Figure. S5b**, **c** offer side- and top-view representations of two vertically stacked intralayer molecular groups in the α-phase; where the orientations of each molecule are labeled as **O1** or **O2**, as shown in **Figure. S5a**. Moreover, we designated the molecule within Layer 2 as "**R′**", which is closest in proximity to the reference molecule "**R**" in Layer 1 and shares the same orientation as **R**. In the α-phase, the **R′** molecule is located at the top left of the **R** molecule (**Figure. S5b**). Interestingly, in the β-structure, a molecule positioned at the corresponding location exhibits a different molecular orientation, as shown in **Figure. S5d-g**. Given that the interconversion of molecular orientations is practically impossible, the result suggests that the molecules in Layer 2 cooperatively shifted by approximately a cell length during the α→β transition. In this regard, the **R′** molecule in the β-phase could be located at the top right of the **R** molecule (**Figure. S5d-g**).

The positional changes of the **R'** molecule relative to the **R** molecule can be summarized as shown in **Figure. S6**. To accomplish the transition, it is believed that a significant barrier was overcome, resulting from the steric hindrance between the side-chains arranged in a zigzag fashion (**Figure. S7**). Therefore, we pinpoint the reconfigurational motions of the side-chain as a molecular aspect that facilitates this transition; see **Figure. 5a** and **Figure. S8**, and **Movie S3**.

Supplementary Tables

Table S1. Single crystal X-ray structures of p-TIPS-DSB at temperatures of 303 – 450 K upon the first heating-cooling cycle.

Name		α-phase, 303 K (Heating)	α-phase, 323 K (Heating)	α-phase, 353 K (Heating)	α-phase, 373 K (Heating)
Empirical formula		C40 H58 O2 Si2	C40 H58 O2 Si2	C40 H58 O2 Si2	C40 H58 O2 Si2
Formula weight		627.04	627.04	627.04	627.04
Temperature		303(2) K	323(2) K	353(2) K	373(2) K
Wavelength		0.700 Å	0.700 Å	0.700 Å	0.700 Å
Crystal system		Triclinic	Triclinic	Triclinic	Triclinic
Space group		*P*-1	*P*-1	*P*-1	*P*-1
Cell Parameters	*a* (Å)	7.9180(16)	7.9390(16)	7.9720(16)	7.9960(16)
	b (Å)	16.114(3)	16.147(3)	16.192(3)	16.230(3)
	c (Å)	16.263(3)	16.256(3)	16.236(3)	16.244(3)
	α (°)	67.65(3)	67.73(3)	67.82(3)	67.86(3)
	β (°)	82.49(3)	82.41(3)	82.29(3)	82.14(3)
	γ (°)	89.03(3)	89.00(3)	88.93(3)	88.81(3)
Volume ($Å^3$)		1901.5(8)	1910.3(8)	1922.0(8)	1933.2(8)
Z		2	2	2	2
Density (Mg/m^3)		1.095	1.090	1.083	1.077
Absorption coefficient (mm^{-1})		0.118	0.118	0.117	0.116
F(000)		684	684	684	684
Crystal size		0.021 × 0.010 × 0.002 mm^3	0.021 × 0.010 × 0.002 mm^3	0.021 × 0.010 × 0.002 mm^3	0.021 × 0.010 × 0.002 mm^3
Theta range for data collection		1.346 to 33.205°	1.343 to 33.056°	1.338 to 32.895°	1.335 to 33.498°
Index ranges		−10<=h<=10, −23<=k<=23 −22<=l<=23	−9<=h<=10, −23<=k<=23, −22<=l<=23	−10<=h<=9, −23<=k<=23, −22<=l<=23	−9<=h<=9, −23<=k<=23, −22<=l<=23
Reflections collected		11333	10785	9173	18317
Independent reflections		11333 [R(int) = ?]	10785 [R(int) = ?]	9173 [R(int) = ?]	9783 [R(int) = 0.0411]
Completeness to theta = 24.835°		92.0 %	91.5 %	91.4 %	92.0 %
Absorption correction		Empirical	Empirical	Empirical	Empirical
Max. and min. transmission		1.000 and 0.916	1.000 and 0.864	1.000 and 0.798	1.000 and 0.779
Refinement method		Full-matrix least-squares on F^2	Full-matrix least-squares on F^2	Full-matrix least-squares on F^2	Full-matrix least-squares on F^2
Data / restraints / parameters		11333 / 0 / 410	10785 / 0 / 410	9173 / 0 / 410	9783 / 0 / 409
Goodness-of-fit on F^2		0.923	0.908	0.870	0.838
Final R indices [I>2sigma(I)]		R_1 = 0.0589 wR_2 = 0.1814	R_1 = 0.0644 wR_2 = 0.1911	R_1 = 0.0709 wR_2 = 0.2001	R_1 = 0.0764 wR_2 = 0.2228
R indices (all data)		R_1 = 0.0955 wR_2 = 0.2030	R_1 = 0.1167 wR_2 = 0.2167	R_1 = 0.1461 wR_2 = 0.2339	R_1 = 0.1557 wR_2 = 0.2652
Extinction coefficient		n/a	n/a	n/a	n/a
Largest diff. peak and hole		0.414 and −0.405 e·$Å^{-3}$	0.373 and −0.317 e·$Å^{-3}$	0.229 and −0.297 e·$Å^{-3}$	0.447 and −0.198 e·$Å^{-3}$

Name		α-phase, 393 K (Heating)	α-phase, 403 K (Heating)	α-phase, 413 K (Heating)	β-phase, 423 K (Heating)
Empirical formula		C40 H58 O2 Si2	C40 H58 O2 Si2	C40 H58 O2 Si2	C40 H58 O2 Si2
Formula weight		627.04	627.04	627.04	627.04
Temperature		393(2) K	403(2) K	413(2) K	423(2) K
Wavelength		0.700 Å	0.700 Å	0.700 Å	0.700 Å
Crystal system		Triclinic	Triclinic	Triclinic	Monoclinic
Space group		*P*-1	*P*-1	*P*-1	$P2_1/c$
Cell Parameters	*a* (Å)	8.0180(16)	8.0350(16)	8.0530(16)	14.838(3)
	b (Å)	16.257(3)	16.277(3)	16.295(3)	8.2000(16)
	c (Å)	16.244(3)	16.240(3)	16.254(3)	16.636(3)
	α (°)	67.93(3)	67.97(3)	68.06(3)	90
	β (°)	81.94(3)	81.83(3)	81.71(3)	99.07(3)
	γ (°)	88.64(3)	88.58(3)	88.49(3)	90
Volume ($Å^3$)		1942.0(8)	1948.0(8)	1956.8(8)	1998.8(7)
Z		2	2	2	2
Density (Mg/m^3)		1.072	1.069	1.064	1.042
Absorption coefficient (mm^{-1})		0.116	0.115	0.115	0.112
F(000)		684	684	684	684
Crystal size		0.021 × 0.010 × 0.002 mm^3	0.021 × 0.010 × 0.002 mm^3	0.021 × 0.010 × 0.002 mm^3	0.021 × 0.010 × 0.002 mm^3
Theta range for data collection		1.346 to 33.344°	1.330 to 33.568°	1.328 to 32.873°	2.442 to 32.680°
Index ranges		−9<=h<=9, −23<=k<=22, −22<=l<=22	−9<=h<=10, −23<=k<=23, −22<=l<=22	−8<=h<=9, −20<=k<=23, 0<=l<=23	−20<=h<=20, −9<=k<=9, −23<=l<=23
Reflections collected		16365	15757	8282	14567
Independent reflections		8871 [R(int) = 0.0399]	8656 [R(int) = 0.0489]	8282 [R(int) = ?]	4725 [R(int) = 0.0477]
Completeness to theta = 24.835°		91.4 %	90.3 %	90.4 %	94.4 %
Absorption correction		Empirical	Empirical	Empirical	Empirical
Max. and min. transmission		1.000 and 0.765	1.000 and 0.761	1.000 and 0.760	1.000 and 0.843
Refinement method		Full-matrix least-squares on F^2	Full-matrix least-squares on F^2	Full-matrix least-squares on F^2	Full-matrix least-squares on F^2
Data / restraints / parameters		8871 / 0 / 409	8656 / 0 / 409	8282 / 0 / 410	4725 / 0 / 200
Goodness-of-fit on F^2		0.857	0.857	0.760	0.945
Final R indices [I>2sigma(I)]		R_1 = 0.0742 wR_2 = 0.2068	R_1 = 0.0780 wR_2 = 0.2054	R_1 = 0.0770 wR_2 = 0.1971	R_1 = 0.1134 wR_2 = 0.3157
R indices (all data)		R_1 = 0.1682 wR_2 = 0.2487	R_1 = 0.1962 wR_2 = 0.2484	R_1 = 0.2082 wR_2 = 0.2527	R_1 = 0.2100 wR_2 = 0.3729
Extinction coefficient		n/a	n/a	n/a	0.023(8)
Largest diff. peak and hole		0.261 and −0.240 $e·Å^{-3}$	0.277 and −0.140 $e·Å^{-3}$	0.299 and −0.169 $e·Å^{-3}$	0.415 and −0.226 $e·Å^{-3}$

Name		β-phase, 433 K (Heating)	β-phase, 450 K (Heating)	β-phase, 433 K (Cooling)	β-phase, 423 K (Cooling)
Empirical formula		C40 H58 O2 Si2	C40 H58 O2 Si2	C40 H58 O2 Si2	C40 H58 O2 Si2
Formula weight		627.04	627.04	627.04	627.04
Temperature		433(2) K	450(2) K	433(2) K	423(2) K
Wavelength		0.700 Å	0.700 Å	0.700 Å	0.700 Å
Crystal system		Monoclinic	Monoclinic	Monoclinic	Monoclinic
Space group		$P2_1/c$	$P2_1/c$	$P2_1/c$	$P2_1/c$
Cell Parameters	*a* (Å)	14.879(3)	14.923(3)	14.875(3)	14.818(3)
	b (Å)	8.2420(16)	8.2800(17)	8.2420(16)	8.2000(16)
	c (Å)	16.631(3)	16.604(3)	16.632(3)	16.625(3)
	α (°)	90	90	90	90
	β (°)	98.95(3)	98.92(3)	98.95(3)	98.99(3)
	γ (°)	90	90	90	90
Volume ($Å^3$)		2014.7(7)	2026.8(7)	2014.3(7)	1995.3(7)
Z		2	2	2	2
Density (Mg/m^3)		1.034	1.027	1.034	1.044
Absorption coefficient (mm^{-1})		0.112	0.111	0.112	0.113
F(000)		684	684	684	684
Crystal size		0.021 × 0.010 × 0.002 mm^3	0.021 × 0.010 × 0.002 mm^3	0.021 × 0.010 × 0.002 mm^3	0.021 × 0.010 × 0.002 mm^3
Theta range for data collection		1.365 to 33.094°	1.360 to 32.299°	1.365 to 33.100°	1.370 to 33.407°
Index ranges		−20<=h<=20, −9<=k<=9, −23<=l<=23	−21<=h<=21, −9<=k<=9, −23<=l<=23	−20<=h<=21, −9<=k<=8, −23<=l<=22	−21<=h<=20, −9<=k<=9, −23<=l<=23
Reflections collected		14471	13748	14482	14190
Independent reflections		4729 [R(int) = 0.0418]	4618 [R(int) = 0.0982]	4736 [R(int) = 0.0435]	4676 [R(int) = 0.0569]]
Completeness to theta = 24.835°		94.4 %	91.3 %	94.2 %	93.3 %
Absorption correction		Empirical	Empirical	Empirical	Empirical
Max. and min. transmission		1.000 and 0.829	1.000 and 0.857	1.000 and 0.815	1.000 and 0.827
Refinement method		Full-matrix least-squares on F^2	Full-matrix least-squares on F^2	Full-matrix least-squares on F^2	Full-matrix least-squares on F^2
Data / restraints / parameters		4729 / 0 / 200	4618 / 71 / 199	4736 / 2 / 200	4676 / 1 / 199
Goodness-of-fit on F^2		0.990	0.993	0.982	0.940
Final R indices [I>2sigma(I)]		R_1 = 0.1182 wR_2 = 0.3385	R_1 = 0.1515 wR_2 = 0.3917	R_1 = 0.1170 wR_2 = 0.3372	R_1 = 0.1168 wR_2 = 0.3275
R indices (all data)		R_1 = 0.2135 wR_2 = 0.3924	R_1 = 0.3032 wR_2 = 0.4535	R_1 = 0.2107 wR_2 = 0.3902	R_1 = 0.2208 wR_2 = 0.3810
Extinction coefficient		0.021(8)	n/a	0.022(8)	n/a
Largest diff. peak and hole		0.387 and −0.258 $e·Å^{-3}$	0.306 and −0.214 $e·Å^{-3}$	0.380 and −0.245 $e·Å^{-3}$	0.377 and −0.233 $e·Å^{-3}$

Name		β-phase, 413 K (Cooling)	β-phase, 403 K (Cooling)	β-phase, 393 K (Cooling)	β-phase, 373 K (Cooling)
Empirical formula		C40 H58 O2 Si2	C40 H58 O2 Si2	C40 H58 O2 Si2	C40 H58 O2 Si2
Formula weight		627.04	627.04	627.04	627.04
Temperature		413(2) K	403(2) K	393(2) K	373(2) K
Wavelength		0.700 Å	0.700 Å	0.700 Å	0.700 Å
Crystal system		Monoclinic	Monoclinic	Monoclinic	Monoclinic
Space group		$P2_1/c$	$P2_1/c$	$P2_1/c$	$P2_1/c$
Cell Parameters	a (Å)	14.788(3)	14.763(3)	14.749(3)	14.725(3)
	b (Å)	8.1610(16)	8.1220(16)	8.0980(16)	8.0490(16)
	c (Å)	16.628(3)	16.624(3)	16.616(3)	16.605(3)
	α (°)	90	90	90	90
	β (°)	99.02(3)	99.00(3)	98.91(3)	98.76(3)
	γ (°)	90	90	90	90
Volume (Å^3)		1981.9(7)	1968.7(7)	1960.6(7)	1945.1(7)
Z		2	2	2	2
Density (Mg/m^3)		1.051	1.058	1.062	1.071
Absorption coefficient (mm^{-1})		0.113	0.114	0.115	0.116
F(000)		684	684	684	684
Crystal size		0.021 × 0.010 × 0.002 mm^3	0.021 × 0.010 × 0.002 mm^3	0.021 × 0.010 × 0.002 mm^3	0.021 × 0.010 × 0.002 mm^3
Theta range for data collection		1.373 to 33.424°	1.375 to 33.029°	1.376 to 32.760°	1.378 to 32.700°
Index ranges		−21<=h<=20, −9<=k<=9, −23<=l<=23	−20<=h<=21, −9<=k<=8, −23<=l<=23	−21<=h<=20, −9<=k<=9, −23<=l<=23	−20<=h<=20, −9<=k<=9, −23<=l<=23
Reflections collected		14068	14001	15717	16538
Independent reflections		4556 [R(int) = 0.0592]	4502 [R(int) = 0.0676]	4874 [R(int) = 0.0508]	4906 [R(int) = 0.0503]
Completeness to theta = 24.835°		93.2 %	92.1 %	95.8 %	96.0 %
Absorption correction		Empirical	Empirical	Empirical	Empirical
Max. and min. transmission		1.000 and 0.836	1.000 and 0.852	1.000 and 0.861	1.000 and 0.865
Refinement method		Full-matrix least-squares on F^2	Full-matrix least-squares on F^2	Full-matrix least-squares on F^2	Full-matrix least-squares on F^2
Data / restraints / parameters		4556 / 1 / 199	4502 / 1 / 199	4874 / 0 / 200	4906 / 0 / 200
Goodness-of-fit on F^2		0.888	0.846	0.911	0.940
Final R indices [I>2sigma(I)]		R_1 = 0.1045 wR_2 = 0.2893	R_1 = 0.1010 wR_2 = 0.2730	R_1 = 0.0909 wR_2 = 0.2773	R_1 = 0.0877 wR_2 = 0.2647
R indices (all data)		R_1 = 0.2020 wR_2 = 0.3488	R_1 = 0.2022 wR_2 = 0.3341	R_1 = 0.1573 wR_2 = 0.3277	R_1 = 0.1400 wR_2 = 0.3151
Extinction coefficient		n/a	n/a	0.037(9)	0.052(10)
Largest diff. peak and hole		0.341 and −0.216 e·Å^{-3}	0.347 and −0.194 e·Å^{-3}	0.625 and −0.221 e·Å^{-3}	0.768 and −0.202 e·Å^{-3}

`Name		β-phase, 353 K (Cooling)	β-phase, 323 K (Cooling)	β-phase, 303 K (Cooling)
Empirical formula		C40 H58 O2 Si2	C40 H58 O2 Si2	C40 H58 O2 Si2
Formula weight		627.04	627.04	627.04
Temperature		353(2) K	323(2) K	303(2) K
Wavelength		0.700 Å	0.700 Å	0.700 Å
Crystal system		Monoclinic	Monoclinic	Monoclinic
Space group		$P2_1/c$	$P2_1/c$	$P2_1/c$
Cell Parameters	*a* (Å)	14.702(3)	14.649(3)	14.647(3)
	b (Å)	8.0130(16)	7.9620(16)	7.9410(16)
	c (Å)	16.588(3)	16.560(3)	16.555(3)
	α (°)	90	90	90
	β (°)	98.63(3)	98.50(3)	98.40(3)
	γ (°)	90	90	90
Volume (Å^3)		1932.0(7)	1910.2(7)	1904.9(7)
Z		2	2	2
Density (Mg/m^3)		1.078	1.090	1.093
Absorption coefficient (mm^{-1})		0.116	0.118	0.118
F(000)		684	684	684
Crystal size		0.021 × 0.010 × 0.002 mm^3	0.021 × 0.010 × 0.002 mm^3	0.021 × 0.010 × 0.002 mm^3
Theta range for data collection		1.380 to 32.689°	2.450 to 33.171°	1.384 to 33.069°
Index ranges		−20<=h<=20, −9<=k<=9, −23<=l<=23	−20<=h<=20, −9<=k<=9, −23<=l<=23	−20<=h<=20, −9<=k<=9, −23<=l<=23
Reflections collected		16999	16513	17473
Independent reflections		5041 [R(int) = 0.0515]	5001 [R(int) = 0.0711]	5217 [R(int) = 0.0658]
Completeness to theta = 24.835°		96.2 %	93.3 %	94.5 %
Absorption correction		Empirical	Empirical	Empirical
Max. and min. transmission		1.000 and 0.879	1.000 and 0.839	1.000 and 0.871
Refinement method		Full-matrix least-squares on F^2	Full-matrix least-squares on F^2	Full-matrix least-squares on F^2
Data / restraints / parameters		5041 / 0 / 200	5001 / 0 / 200	5217 / 0 / 200
Goodness-of-fit on F^2		0.958	0.977	1.007
Final R indices [I>2sigma(I)]		R_1 = 0.0905 wR_2 = 0.2694	R_1 = 0.1006 wR_2 = 0.2795	R_1 = 0.0985 wR_2 = 0.2795
R indices (all data)		R_1 = 0.1391 wR_2 = 0.3160	R_1 = 0.1557 wR_2 = 0.3269	R_1 = 0.1442 wR_2 = 0.3238
Extinction coefficient		0.084(14)	0.13(2)	0.14(2)
Largest diff. peak and hole		0.852 and −0.268 e·Å^{-3}	0.818 and −0.298 e·Å^{-3}	0.948 and −0.293 e·Å^{-3}

Table S2. Summary of unit cell parameters for the p-TIPS-DSB single crystal structures at different temperatures.

Phase	Temperature		*a* (Å)	*b* (Å)	*c* (Å)	*α* (°)	*β* (°)	*γ* (°)	V (Å^3)	CCDC
α	303 K, Heating	PASCal Input	7.92	16.11	16.26	67.65	82.49	89.03	1901.45	2346778
	323 K, Heating		7.94	16.15	16.26	67.73	82.41	89.00	1910.33	2346779
	353 K, Heating		7.97	16.19	16.26	67.82	82.29	88.93	1921.99	2346780
	373 K, Heating		8.00	16.23	16.24	67.86	82.14	88.81	1933.20	2346781
	393 K, Heating		8.02	16.26	16.24	67.93	81.94	88.64	1941.88	2346782
	403 K, Heating		8.04	16.28	16.24	67.97	81.83	88.58	1947.96	2346783
	413 K, Heating		8.05	16.30	16.25	68.06	81.71	88.49	1956.88	2346784
β	423 K, Heating	x	14.84	8.20	16.64	90.00	99.07	90.00	1998.82	2346785
	433 K, Heating	x	14.88	8.24	16.63	90.00	98.95	90.00	2014.67	2346786
	450 K, Heating	PASCal Input	14.92	8.28	16.60	90.00	98.92	90.00	2026.82	2346787
	433 K, Cooling		14.88	8.24	16.63	90.00	98.95	90.00	2014.25	2346788
	423 K, Cooling		14.82	8.20	16.63	90.00	98.99	90.00	1995.25	2346789
	413 K, Cooling		14.79	8.16	16.63	90.00	99.02	90.00	1981.93	2346790
	403 K, Cooling		14.76	8.12	16.62	90.00	99.00	90.00	1968.76	2346791
	393 K, Cooling		14.75	8.10	16.62	90.00	98.91	90.00	1960.62	2346792
	373 K, Cooling		14.73	8.05	16.61	90.00	98.76	90.00	1945.09	2346793
	353 K, Cooling		14.70	8.01	16.59	90.00	98.63	90.00	1932.06	2346794
	323 K, Cooling		14.65	7.96	16.56	90.00	98.50	90.00	1910.27	2346795
	303 K, Cooling		14.65	7.94	16.56	90.00	98.40	90.00	1904.89	2346796

Table S3. Reported TE coefficients for the representative organic crystals.

Material	T range (K)	TE coefficient (MK^{-1}) α_{X1}	α_{X2}	α_{X3}	α_V	Reference
p-TIPS-DSB	α form 303–413 β form 303–450	α form -38.6 β form 10.2	α form 116.6 β form 130.7	α form 175.0 β form 291.7	α form 255.3 β form 444.9	This work
diazo-I	280–320	- 65	64	211	210	(12)
1N’	100–310	- 189	151	255	217	(13)
(S,S)-octa-3,5-diyn-2,7-diol[a]	240–330	$156 < \alpha_a < 515$	$-32 < \alpha_b < -85$	$-48 < \alpha_c < -204$	$47.5 < \alpha_v < 241$	(14)
1,4-bis(1'-hydroxycyclopentyl)-1,3-butadiyne	100–340	- 116	118	184.2	185.2	(15)
TI form II	249–400	- 19.8	28.2	154.7	162.2	(16)
ABN	100–160	10.9	54.8	174.7	250	(17)
BNDI-T	100–375	452	- 16.8	- 154	273	(18)
Ba-bpe	190–290	18	65	115	200	(19)
res, (AP)0.75, (BPE)0.25	190–290	- 10	82	136	210	(20)
(ClADA)(18C6) ClO_4	333–378	- 22.5	380.2	- 45.6	311.2	(21)
Guanidinium nitrate	100–294	4.98	6.81	211.46	210.79	(22)
DSBr	293–473	-8.94	34.6	281.6	314.5	(23)
IMACET-D_6	270-400	246.2	291.7	- 214.7	367.6	(24)

a. Their TE coefficients were based on the direction of unit cell axis.

Supplementary Figures

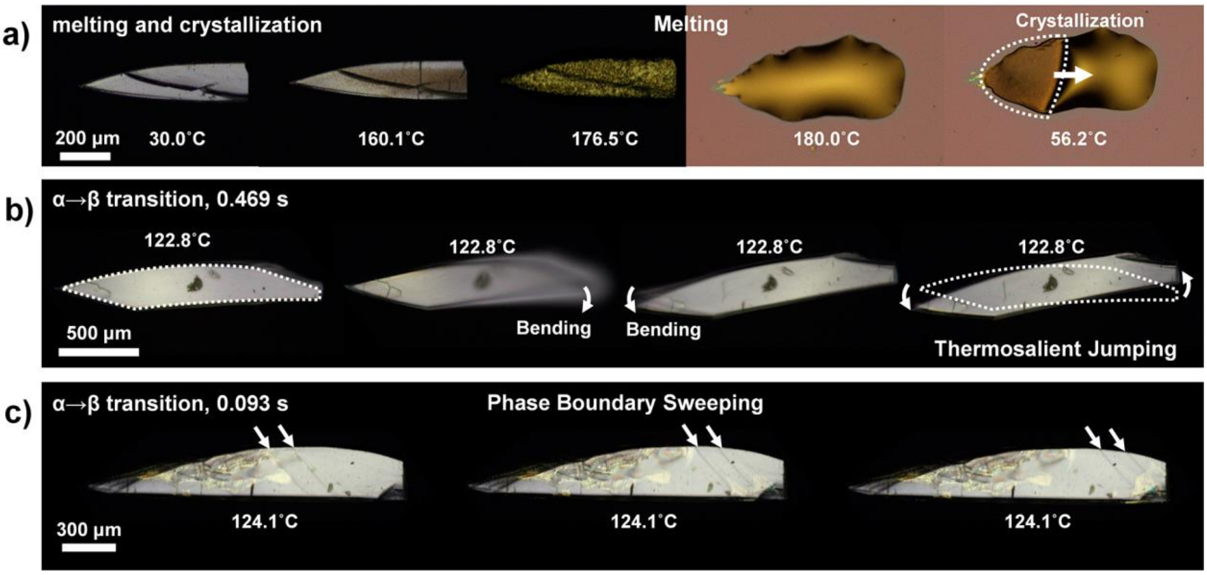

Figure. S1. a) VT-CPOM images corroborating the melting and crystallization of the p-TIPS-DSB crystal. b) Thermosalient jumping, c) and phase boundary sweeping of the p-TIPS-DSB occurred upon α→β transition.

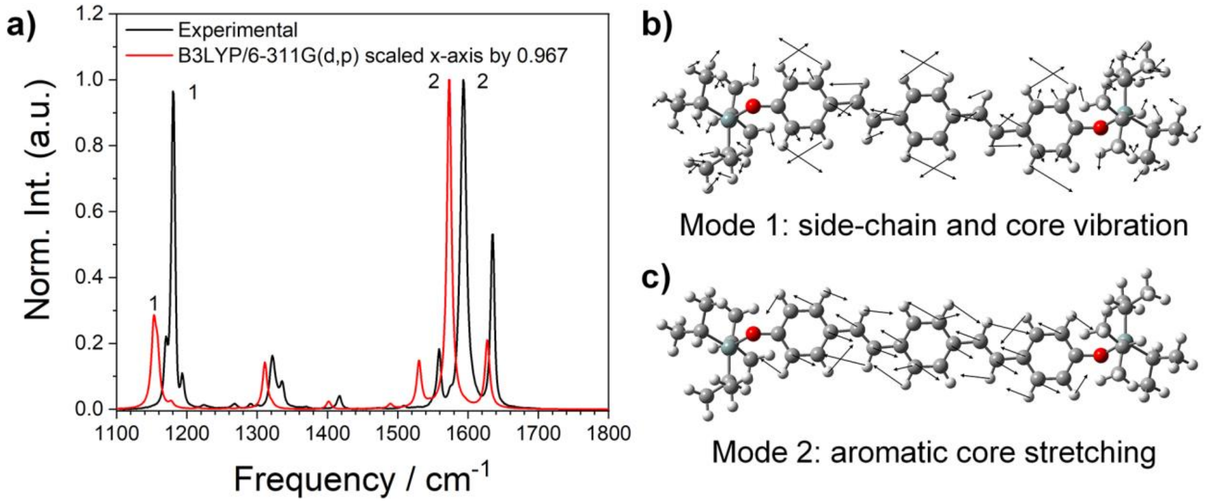

Figure. S2. a) Experimental (black line) and DFT calculated Raman (red line) spectra with the denotation of Modes 1 and 2 of **Figure. 4**. Vibrational motions of Modes 1 (b) and 2 (c), simulated through DFT calculation.

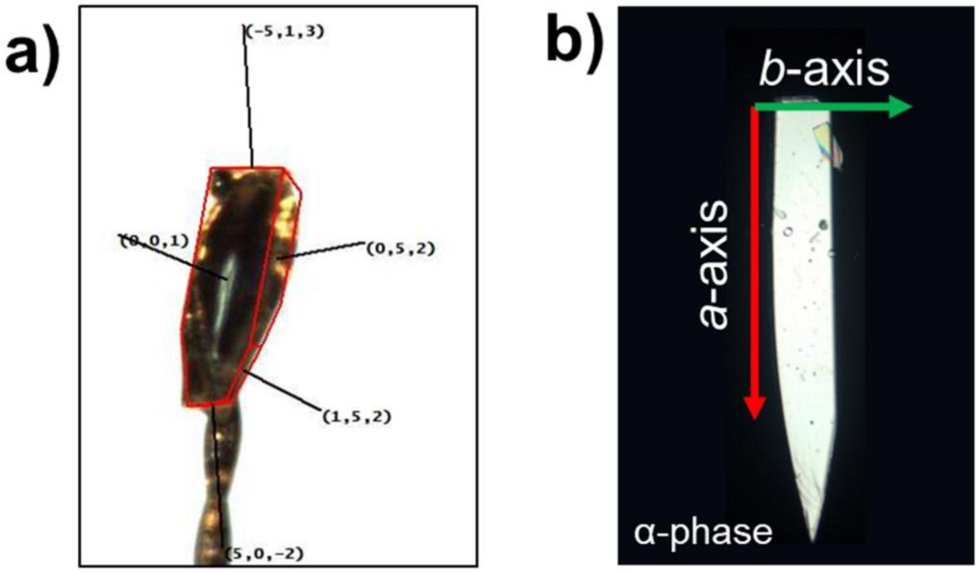

Figure. S3. a) Habit plane analysis of the α-phase p-TIPS-DSB crystal. b) The result indicates that side facets are (0 5 2) and (0 -5 -2), thereby pointing out that the long-axis of the crystal is aligned with the crystallographic *a*-axis.

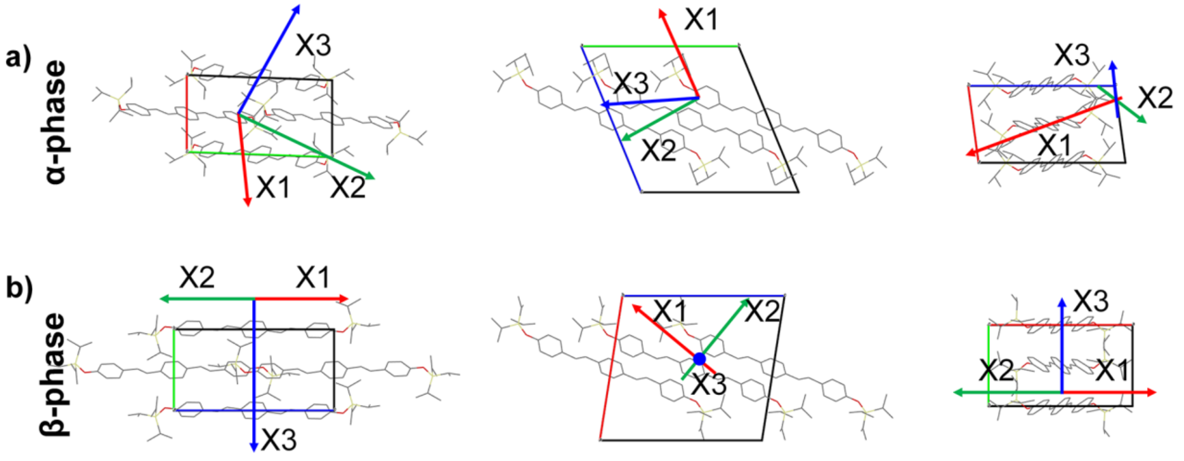

Figure. S4. The orientation of the X1 – X3 axes in a) α- and b) β-phases determined through PASCal, viewed from each crystallographic axis perspective.

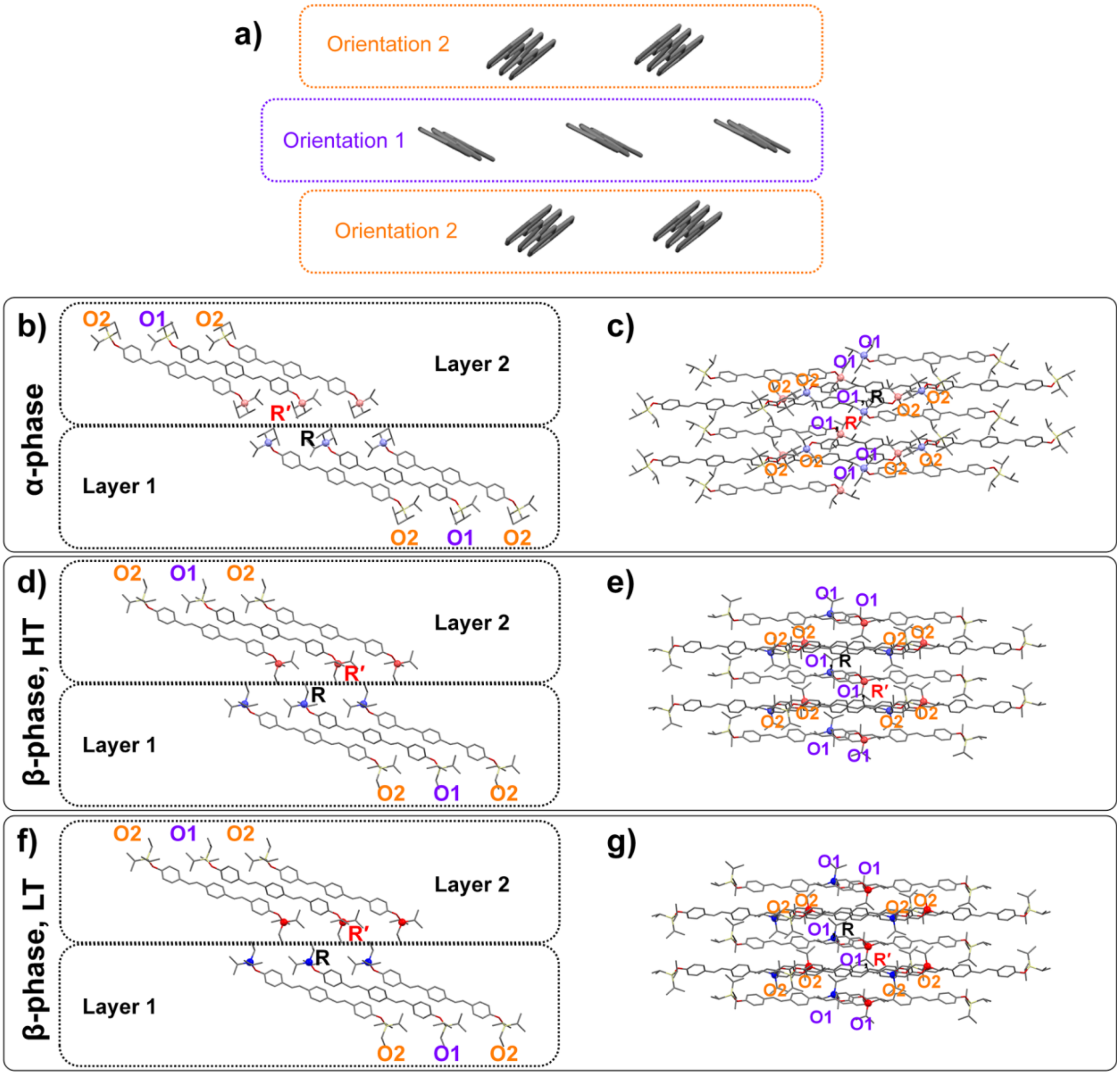

Figure. S5. a) Two distinct molecular orientations found in herringbone style p-TIPS-DSB structure. b, c) Side- and top-view representations of two vertically stacked intralayer molecular groups in the α-phase (30 °C), d, e) β-phase (150 °C), f, g) and β-phase (30 °C). In the figures, two different orientations are denoted as O1 and O2. Moreover, the reference molecule in Layer 1 is denoted as “R” and the molecule within Layer 2 that is closest in with the same orientation as “R” is denoted as “R'.”

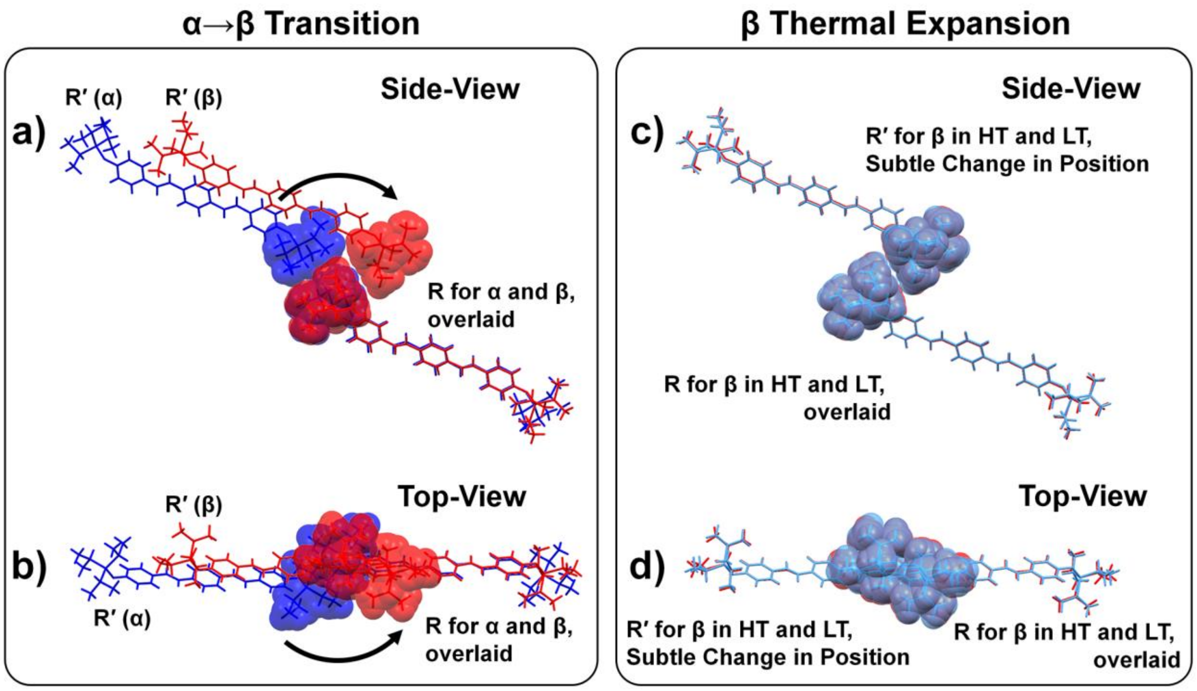

Figure. S6. a, b) The side- and top-view images showing a substantial positional change of R' respect to R molecule upon the α→β transition. The blue and red molecules represent α- and β-phase, respectively. c, d) The side- and top-view images showing interlayer shear does not occur upon thermal expansion. The red and sky-blue molecules represent the β-phase at HT and LT conditions, respectively.

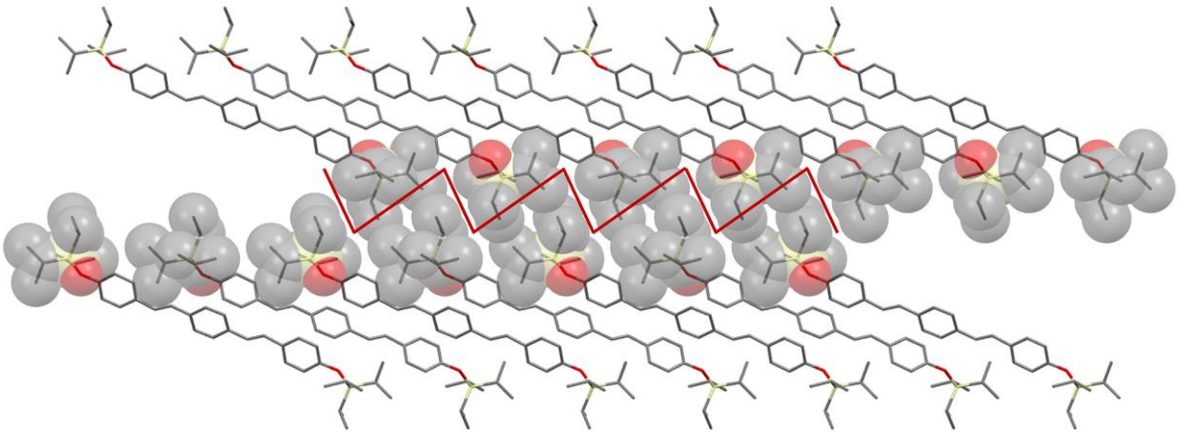

Figure. S7. The zigzag arrangement of interlayer TIPS units resulting in considerable steric hindrance for interlayer shear. The β-structure at 30 °C is adopted for schematic illustration.

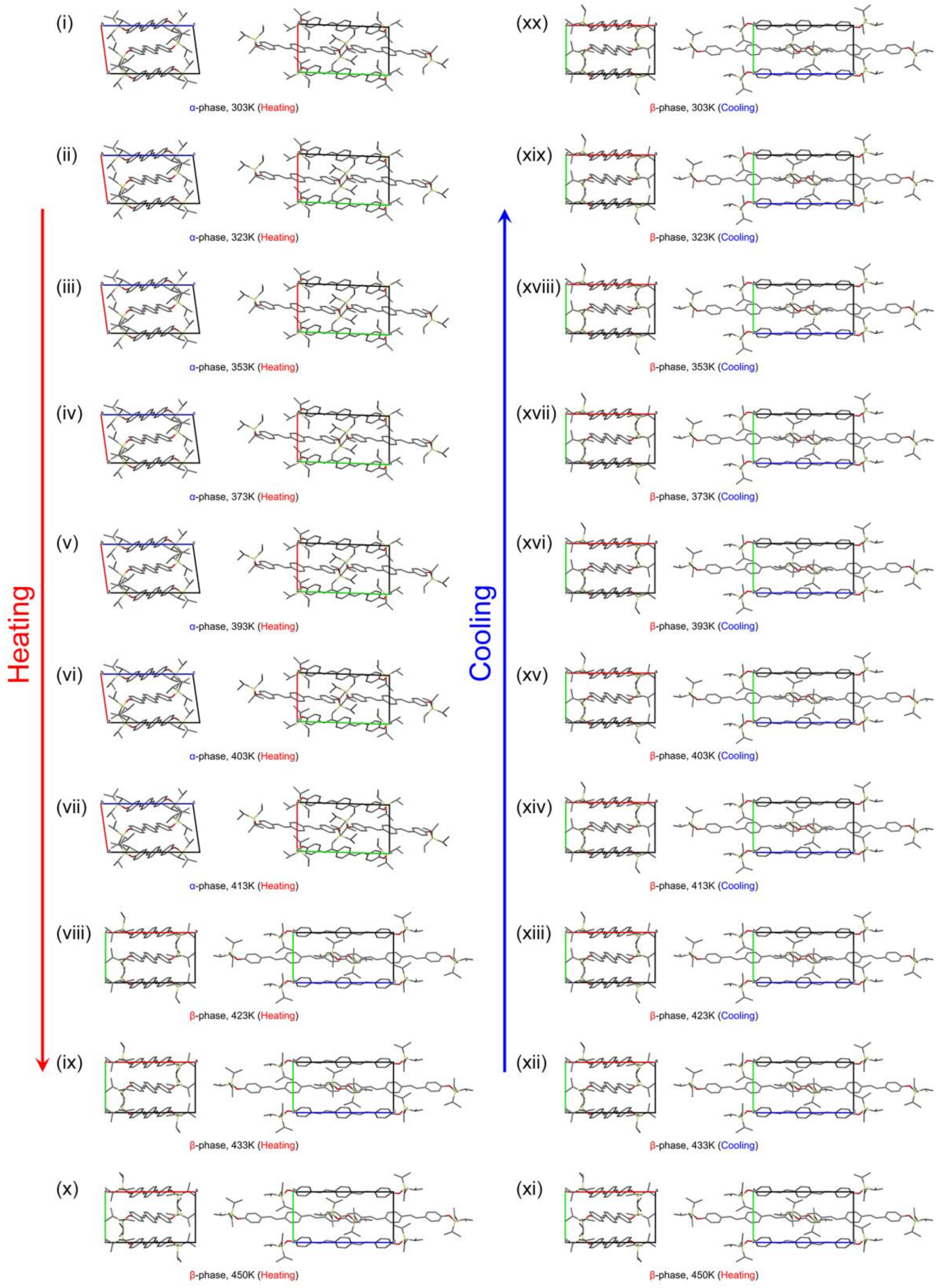

Figure. S8. The changes in structure and side-chain orientation with respect to temperature. Each image is integrated to form **Movie S3**, and their rotational angles are presented in **Figure. 5b** measured through the torsional angles analysis.

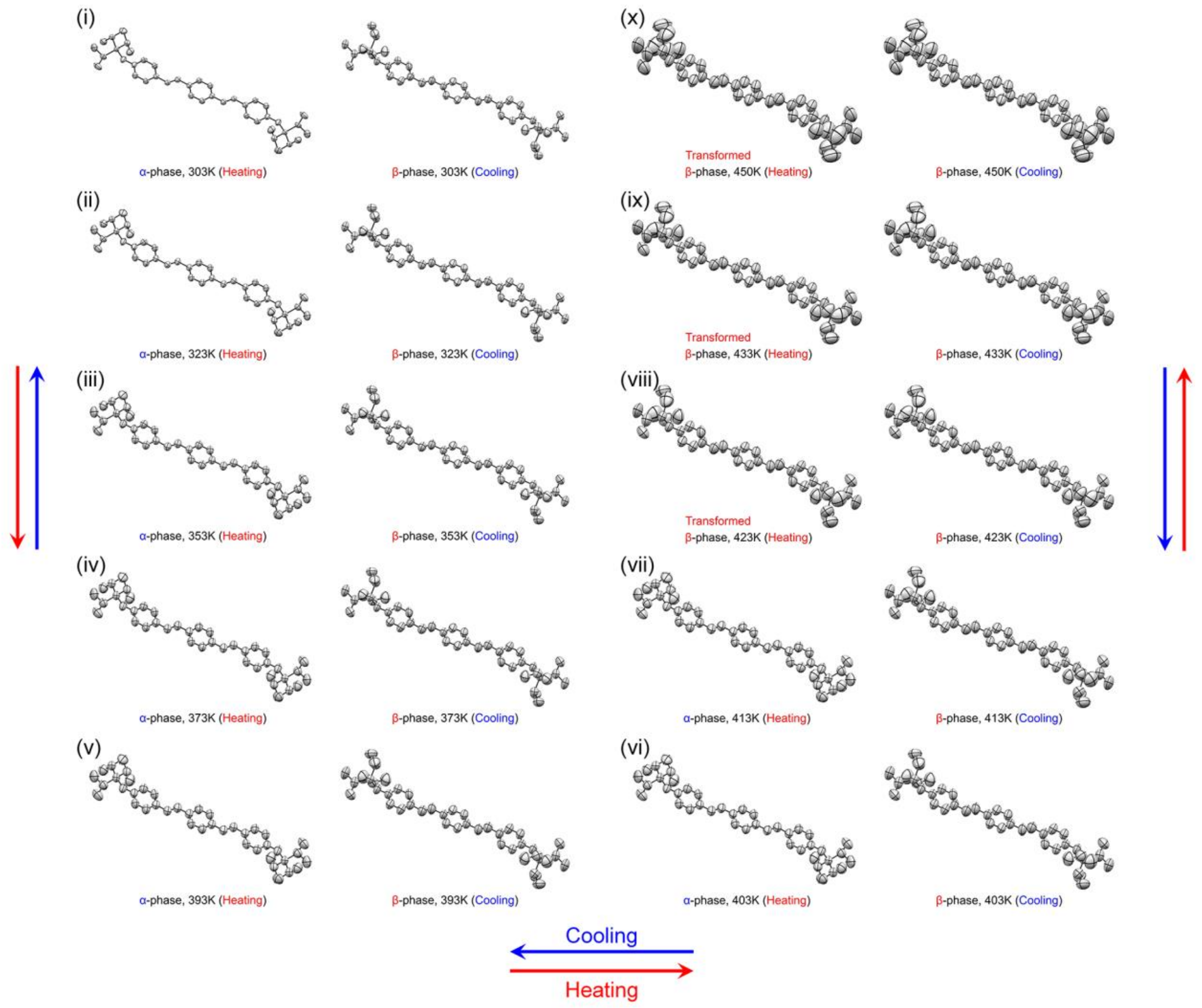

Figure. S9. Thermal ellipsoid plots for α- and β-phases with respect to temperature. For an easy comparison of atomic displacement between the two phases at the same temperature, they are arranged side by side. These images are consolidated to form **Movie S4**.

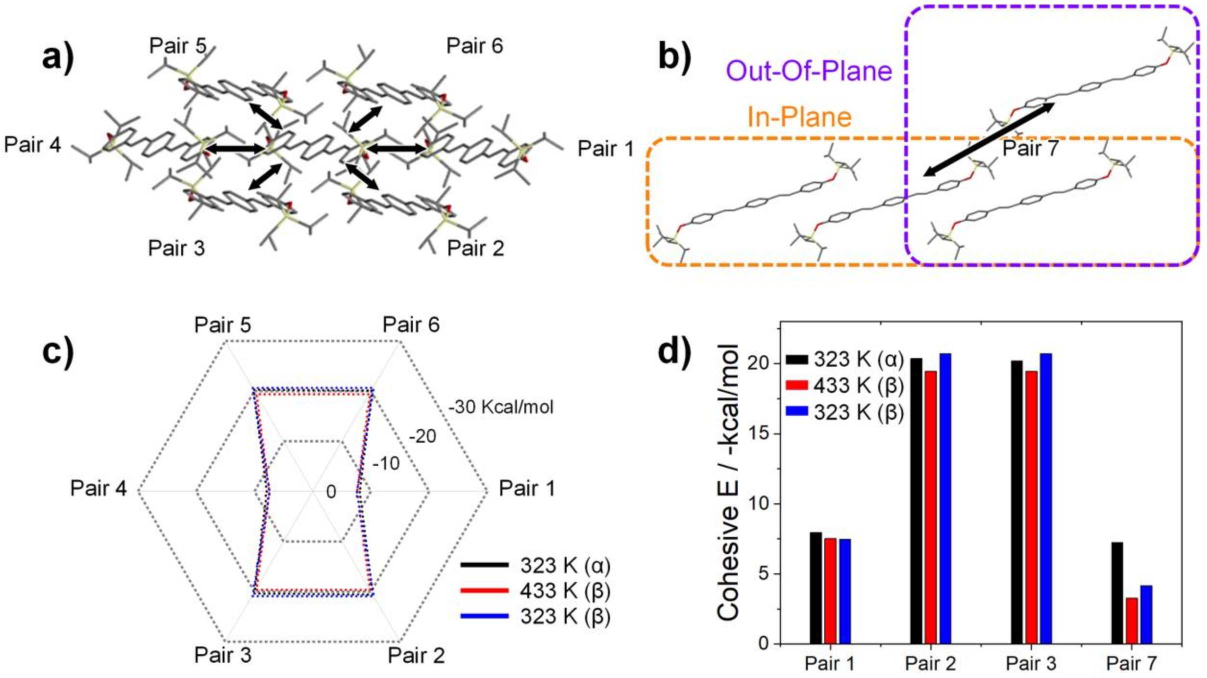

Figure. S10. Defined molecular pairs in the in-plane (a: intraplane) and out-of-plane (b: interplane) directions, where the latter is defined by the nearest proximity of TIPS units. c, d) The DFT calculated cohesive energies of the α-phase at 50 °C (black), β-phase at 160 °C (red), and β-phase at 50 °C (blue).

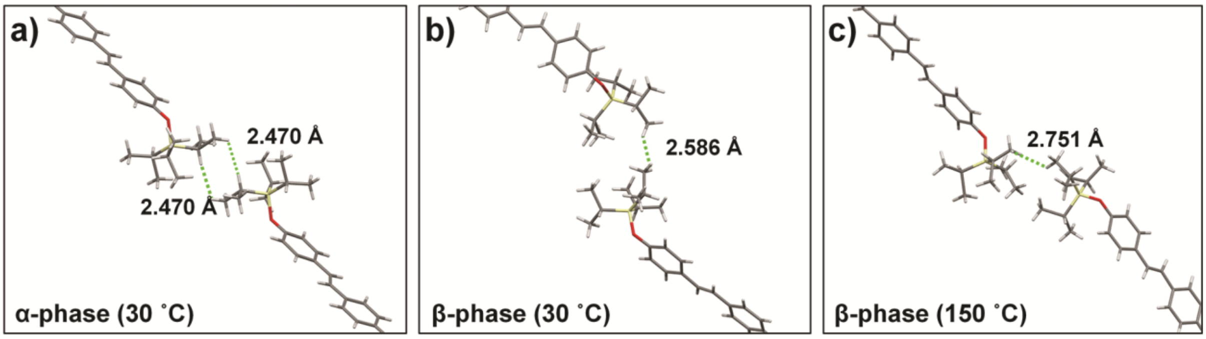

Figure. S11. The shortest –CH···HC– contact distance between interlayer side-chains for a) the α-phase at 30°C, b) β-phase at 30 °C, and c) β-phase at 150 °C, corroborated from the SCXRD results.

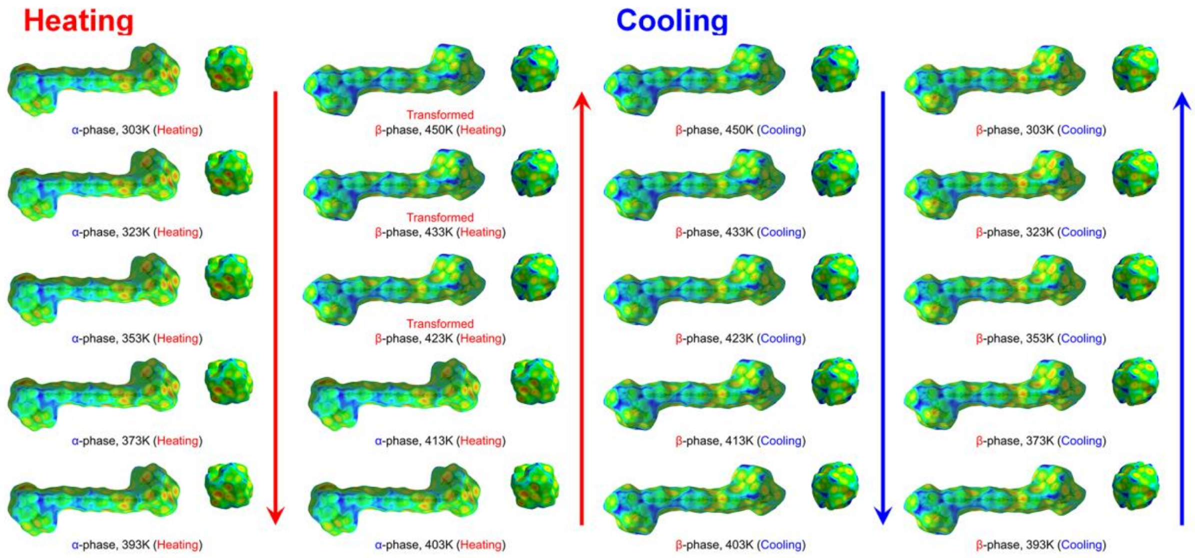

Figure. S12. d_e plots calculated based on the Crystal Explorer software at each temperature, applying the same scale (1.0–2.4 Å). The images are integrated to form **Movie S5**.

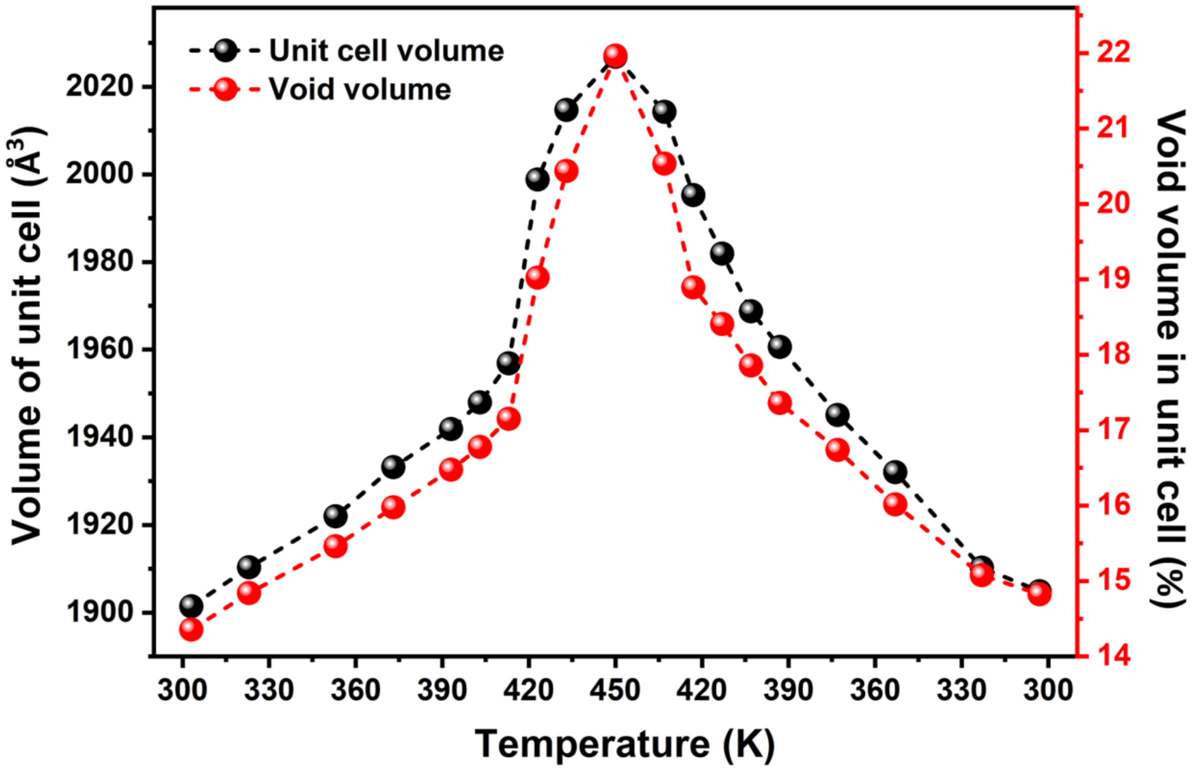

Figure. S13. The ratios of void volume to cell volume (red). The cell volumes are also shown in black for comparison.

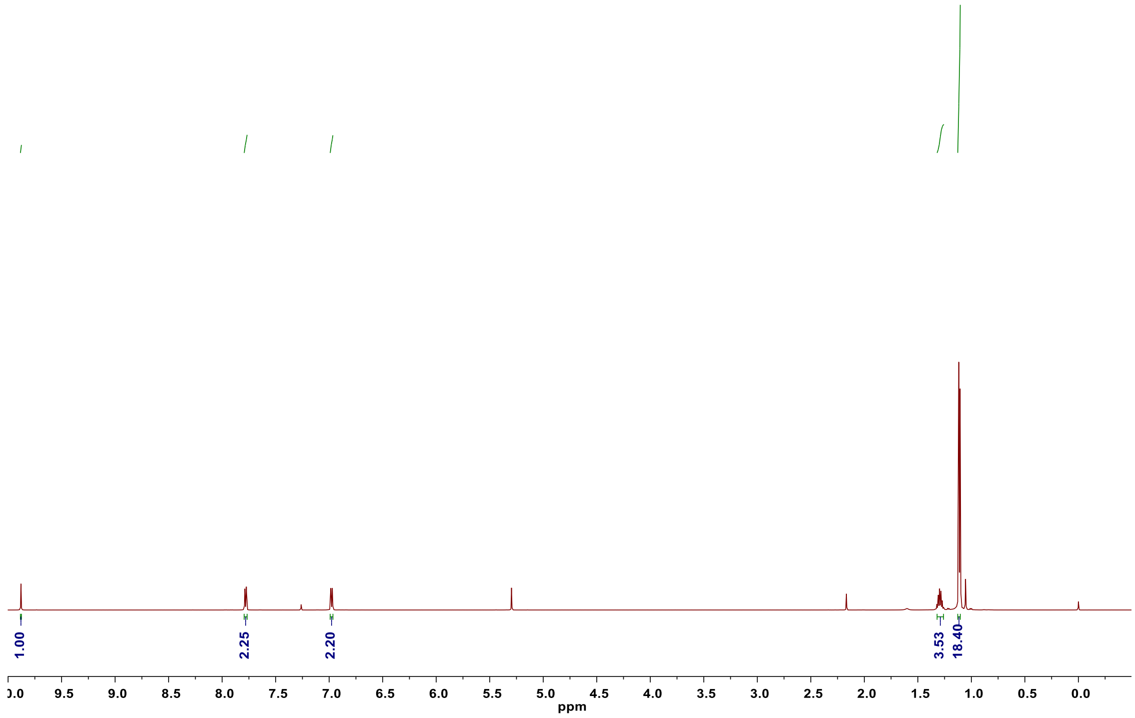

Figure. S14. ^{1}H-NMR spectrum of 4-((triisopropylsilyl)oxy)benzaldehyde (2) (600 MHz, $CDCl_3$) : 9.88 (s, 1H), 7.77 (d, 2H), 6.97 (d, 2H), 1.25-1.32 (m, 3H), 1.10 (m, 18H) ppm.

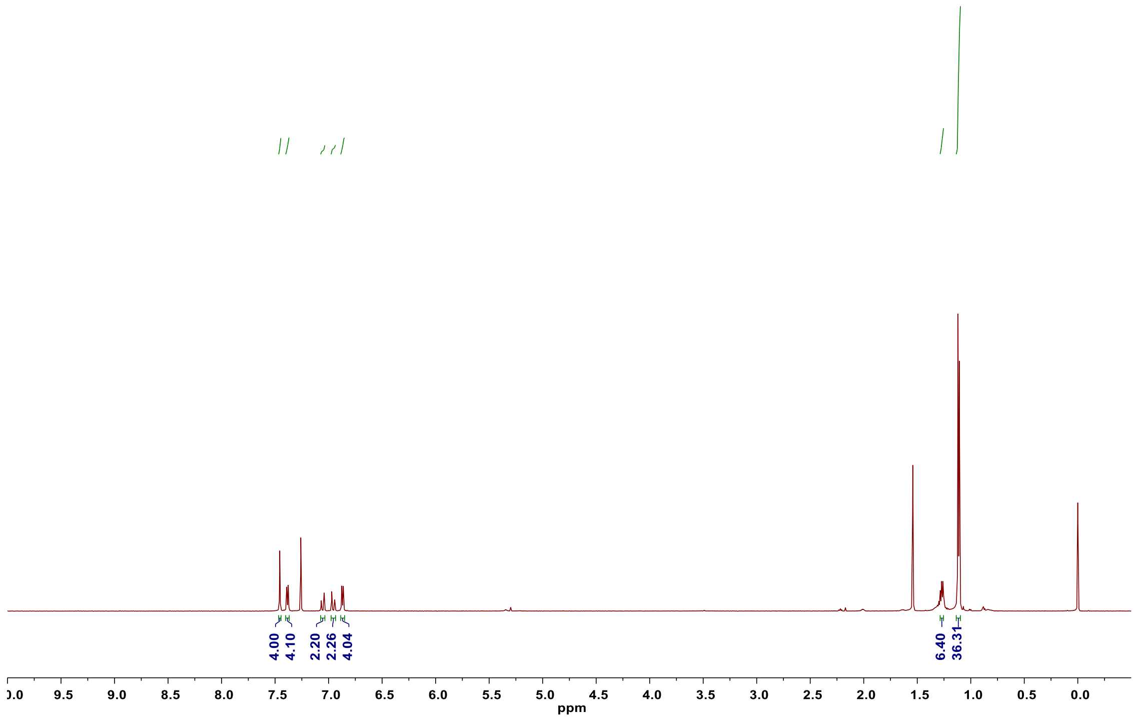

Figure. S15. ^{1}H-NMR spectrum of p-TIPS-DSB (3) (600 MHz, $CDCl_3$) : 7.45 (s, 4H), 7.38 (d, 4H), 7.04 (d, 2H), 6.94 (d, 2H), 6.86 (d, 4H), 1.26 (m, 6H), 1.11 (m, 36H) ppm.

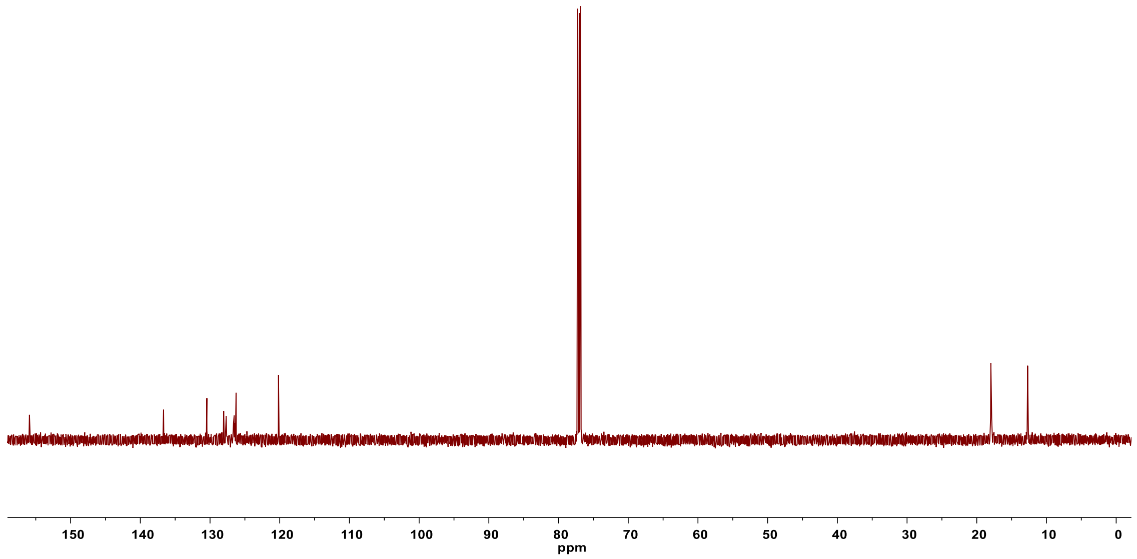

Figure. S16. ^{13}C-NMR spectrum of p-TIPS-DSB (3) (600 MHz, $CDCl_3$) : 155.92, 136.67, 130.46, 128.13, 127.77, 126.27, 120.15, 17.94, 12.70.

Captions for Supplementary Videos

Movie S1. Thermosalient jumping of p-TIPS-DSB single crystal upon the α→β transition.

Movie S2. Phase boundary sweeping behavior of p-TIPS-DSB single crystal upon the α→β transition.

Movie S3. Reorientation of side-chains with respect to temperature observed from VT-SCXRD study.

Movie S4. Thermal ellipsoid plots with respect to temperature observed from VT-SCXRD study.

Movie S5. Simulated d_e mapping on Hirshfeld surface with respect to temperature.

Movie S6. The p-TIPS-DSB single crystal actuator pushing a glass piece of lower weight (748.2 mg).

Movie S7. The p-TIPS-DSB single crystal actuator pushing a glass piece of higher weight (1010.1 mg).

Movie S8. The amplified motion of the p-TIPS-DSB single crystal actuator by incorporating a 1:15 lever.

Reference

[1] J. J. McKinnon, D. Jayatilaka, M. A. Spackman, *Chem. Commun.* **2007**, 3814-3816.
[2] M. J. Cliffe, A. L. Goodwin, *J. Appl. Cryst.* **2012**, *45*, 1321-1329.
[3] J. W. Shin, K. Eom, D. Moon, *J. Synchrotron Radiat.* **2016**, *23*, 369-373.
[4] Z. Otwinowski, W. Minor, *Methods Enzymol* **1997**, *276*, 307-326.
[5] G. M. Sheldrick, *Acta Crystallogr. Sect. A* **2015**, *71*, 3-8.
[6] G. M. Sheldrick, *Acta Crystallogr. Sect. C* **2015**, *71*, 3-8.
[7] A. L. Spek, *Acta Cryst. C.* **2015**, *71*, 9-18.
[8] J. P. Perdew, K. Burke, M. Ernzerhof, *Phys. Rev. Lett.* **1996**, *77*, 3865-3868.
[9] S. Grimme, *J. Comput. Chem.* **2006**, *27*, 1787-1799.
[10] T. Hirano, in *MOPAC Manual*, Seventh Edition ed. (Ed.: J. J. P. Stewart), **1993**.
[11] S. Yu, S. Yang, M. Cho, *J. Appl. Phys.* **2013**, *114*.
[12] X. Ding, D. K. Unruh, L. Ma, E. J. van Aalst, E. W. Reinheimer, B. J. Wylie, K. M. Hutchins, *Angew. Chem. Int. Ed.* **2023**, *62*, e202306198.
[13] S. Dutta, P. Munshi, *J. Phys. Chem. C* **2020**, *124*, 27413-27421.
[14] D. Das, T. Jacobs, L. J. Barbour, *Nat. Mater.* **2010**, *9*, 36-39.
[15] D. Das, L. J. Barbour, *CrystEngComm* **2018**, *20*, 5123-5126.
[16] A. Janiak, C. Esterhuysen, L. J. Barbour, *Chem. Commun.* **2018**, *54*, 3727-3730.
[17] L. O. Alimi, D. P. van Heerden, P. Lama, V. J. Smith, L. J. Barbour, *Chem. Commun.* **2018**, *54*, 6208-6211.
[18] M. Dharmarwardana, B. M. Otten, M. M. Ghimire, B. S. Arimilli, C. M. Williams, S. Boateng, Z. Lu, G. T. McCandless, J. J. Gassensmith, M. A. Omary, *Proc. Natl. Acad. Sci. U.S.A.* **2021**, *118*, e2106572118.
[19] N. Juneja, D. K. Unruh, K. M. Hutchins, *Chem. Mater.* **2023**, *35*, 7292-7300.
[20] X. Ding, D. K. Unruh, R. H. Groeneman, K. M. Hutchins, *Chem. Sci.* **2020**, *11*, 7701-7707.
[21] S. Y. Ge, R. K. Huang, J. B. Wu, K. Takahashi, C. S. Lee, T. Nakamura, *Chem. Mater.* **2023**, *35*, 4311-4317.
[22] D. P. Karothu, R. Ferreira, G. Dushaq, E. Ahmed, L. Catalano, J. M. Halabi, Z. Alhaddad, I. Tahir, L. Li, S. Mohamed, M. Rasras, P. Naumov, *Nat. Commun.* **2022**, *13*, 2823.
[23] D. Manoharan, S. Ranjan, F. Emmerling, B. Bhattacharya, S. Takamizawa, S. Ghosh, *J. Mater. Chem. C* **2024**, *12*, 2515-2525.
[24] M. K. Panda, R. Centore, M. Causa, A. Tuzi, F. Borbone, P. Naumov, *Sci. Rep.* **2016**, *6*, 29610.
